# Computational mechanistic study of the unimolecular dissociation of ethyl hydroperoxide and its bimolecular reactions with atmospheric species

**DOI:** 10.1038/s41598-020-71881-3

**Published:** 2020-09-14

**Authors:** Mansour H. Almatarneh, Asmaa Alnajajrah, Mohammednoor Altarawneh, Yuming Zhao, Mohammad A. Halim

**Affiliations:** 1 Department of Chemistry, University of Jordan, Amman, 11942 Jordan; 2 Department of Chemistry, Memorial University, St. John’s, NL A1B 3X7 Canada; 3 Department of Chemical and Petroleum Engineering, United Arab Emirates University, Al-Ain, 15551 UAE; 4 Department of Physical Sciences, University of Arkansas-Fort Smith, Fort Smith, AR 72913 USA

**Keywords:** Chemistry, Theoretical chemistry, Computational chemistry

## Abstract

A detailed computational study of the atmospheric reaction of the simplest Criegee intermediate CH_2_OO with methane has been performed using the density functional theory (DFT) method and high-level calculations. Solvation models were utilized to address the effect of water molecules on prominent reaction steps and their associated energies. The structures of all proposed mechanisms were optimized using B3LYP functional with several basis sets: 6-31G(d), 6-31G (2df,p), 6-311++G(3df,3pd) and at M06-2X/6-31G(d) and APFD/6-31G(d) levels of theory. Furthermore, all structures were optimized at the B3LYP/6-311++G(3df,3pd) level of theory. The intrinsic reaction coordinate (IRC) analysis was performed for characterizing the transition states on the potential energy surfaces. Fifteen different mechanistic pathways were studied for the reaction of Criegee intermediate with methane. Both thermodynamic functions (Δ*H* and Δ*G*), and activation parameters (activation energies E_a_, enthalpies of activation ΔH^ǂ^, and Gibbs energies of activation ΔG^ǂ^) were calculated for all pathways investigated. The individual mechanisms for pathways **A1**, **A2**, **B1**, and **B2**, comprise two key steps: (i) the formation of ethyl hydroperoxide (EHP) accompanying with the hydrogen transfer from the alkanes to the terminal oxygen atom of CIs, and (ii) a following unimolecular dissociation of EHP. Pathways from **C1 → H1** involve the bimolecular reaction of EHP with different atmospheric species. The photochemical reaction of methane with EHP (pathway **E1**) was found to be the most plausible reaction mechanism, exhibiting an overall activation energy of 7 kJ mol^−1^, which was estimated in vacuum at the B3LYP/6-311++G(3df,3pd) level of theory. All of the reactions were found to be strongly exothermic, expect the case of the sulfur dioxide-involved pathway that is predicted to be endothermic. The solvent effect plays an important role in the reaction of EHP with ammonia (pathway **F1**). Compared with the gas phase reaction, the overall activation energy for the solution phase reaction is decreased by 162 and 140 kJ mol^−1^ according to calculations done with the SMD and PCM solvation models, respectively.

## Introduction

Methane (CH_4_) has several appealing properties; it is an unscented, colorless gas that is discharged from both natural and anthropogenic resources and gives energy or heat via the combustion process. The abundance of methane makes it widely used in energy recovery systems, transport, and heating. Be that as it may, the emission of methane into the atmosphere has negative consequences as it is considered a greenhouse gas with approximately 20 times the effect of carbon dioxide^1^. Combustion and farming-related activities account for most of the reported methane emissions (63%, or 566 Tg CH_4_/year)^2^. Human methane wastes are produced by the utilization of petroleum products, landfilling, livestock farming, and biomass burning. Natural sources of methane are wetlands, oceans, rivers, lakes, permafrost, gas hydrates, geological sources (marine and terrestrial), wildfires, vegetation, and wild animals. The proportion of human to natural methane creation has expanded relentlessly since the advent of the industrial revolution. With increased food requirements, greater waste generation, and greater use of fossil fuels by an increasing human population, the trend is continuously growing. The favored utilization of methane as a fuel is primarily because its combustion is exceedingly exothermic (Δ*H* =  − 891 kJ mol^−1^)^2^. Methane plays a key role in the chemistry of both the stratosphere and the troposphere^3^. It importantly affects the groupings of ozone and water vapor, and on the change of responsive chlorine to less receptive HCl in the stratosphere^4^. Methane and ozone (O_3_) are chemically active climate agents. It merits referencing that climate-chemistry interactions have a significant impact on the two compounds. Ozone, which is an auxiliary compound in the environment, is present in the troposphere and stratosphere, and it is separated through changes in the barometrical conveyance. Methane is an essential compound emitted from various sources as mentioned before, while atmospheric as well as oxidative decomposition of methane can be initiated by hydroxyl radicals (OH)^5^.

The reactions of the chemically active atmosphere gases (e.g., methane and ozone) continue to reshape the chemical composition and reactivity of other important species in the atmosphere. The title reaction also influences other fundamentally important phenomena in the atmosphere, namely, solar radiation, temperature gradients, and air dynamics. They are expected to play an important role in processes determining the interactions between the atmosphere and the biosphere. The chemistry of methane is likewise influencing the atmosphere through its impact on ozone. In addition, outflows of air toxins, which determine regional air quality by interacting with ozone and methane, have the likelihood to change the climate^3,6^. Methane assumes a significant role in the global carbon cycle and energy utilization and exerts important effects on atmospheric chemistry and climate. As a result of the constant growth of methane emissions every year, it has received more and more attention and concerns. It is worth mentioning that the atmospheric methane has now reached 256% of its pre-industrial level because of expanded discharges from human resources as per the reports of the World Meteorological Organization (WMO)^7^. Methane and other gases, such as ethane, carbon dioxide, ozone, and nitrous oxide, are the major contributors to greenhouse gas^1^. Some halocarbons are viewed as strong greenhouse gases and are related to stratospheric ozone exhaustion. Photochemical processes that prompt the formation of particulates and secondary photo-oxidants such as ozone in the environment lead to photochemical smog, which happens with the commitment both of monoterpene and hydrocarbons^8^. Atmospheric trace gases with more than 500 species have been identified in various parts of the atmosphere. Thermodynamic and dynamic investigations indicate that the rate-determining step of the oxidation of methane is the abstraction of hydrogen from the C–H bond by potent oxidizing agents in the atmosphere, for example, OH, NO/NO_2_ and NO_3_^9^. Oxidation of hydrocarbons is the major source for the production of a large array of pollutants, namely polycyclic aromatic hydrocarbons (PAHs), O_3_, CO, and aerosols. Without nitrogen oxides, hydrocarbons are oxidized at the *ppm* level in the air. Alkyl hydroperoxides are formed at the early stages of the oxidation sequences. The presence of peroxy acids during the oxidation of methane was also confirmed^10^. Alkene ozonolysis is often regarded as a significant climatic sink for the tropospheric alkenes^7^.

**Figure 1:**
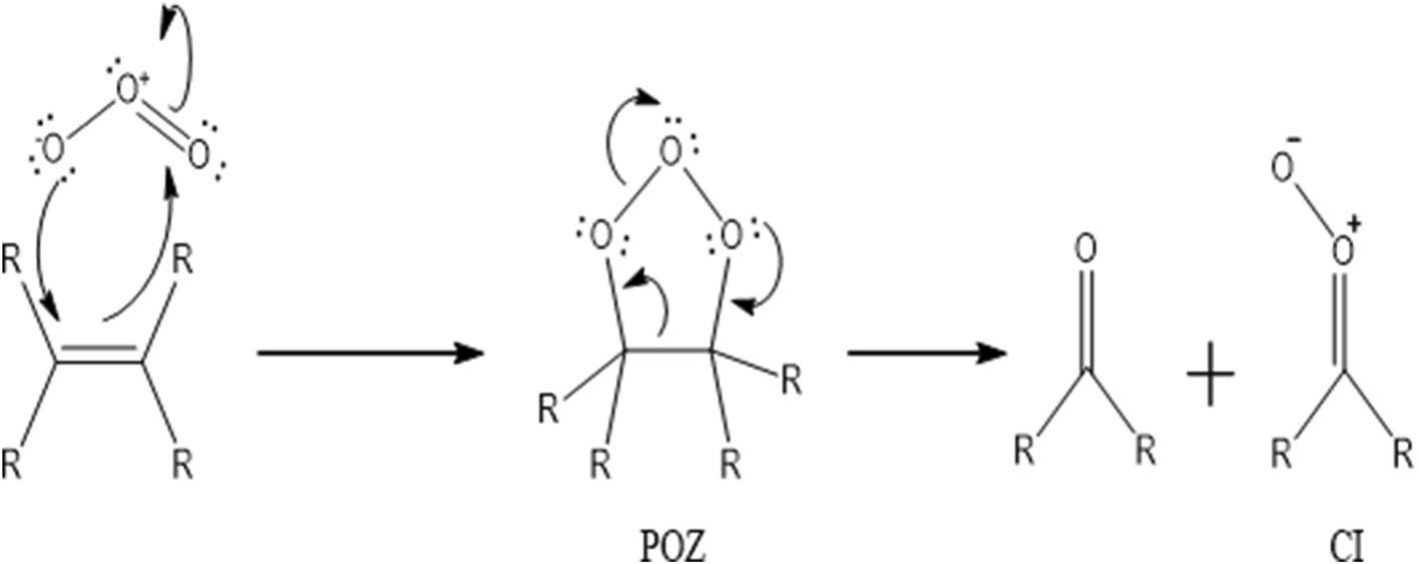
General mechanism of alkene ozonolysis.

The mechanism of ozonolysis was broadly investigated owing to the significance of solution-phase ozonolysis in synthetic chemistry. Almatarneh et al. computationally examined the ozonolysis and related reactions of various compounds (phenanthrene, sabinene and monoterpenes, methylbutenol, β-pinene, and methyl vinyl ketone oxide) at various levels of theory^11–19^. Rudolf Criegee proposed the mechanism of alkene ozonolysis in 1949, which involves the formation of a carbonyl compound and a carbonyl oxide intermediate. The carbonyl oxide is a zwitterion that is known as the Criegee intermediate (CI)^20^. CIs are significant intermediates in the environment, where they play a major role in the formation of hydroxyl radicals and different organic compounds. The initial step in the ozonolysis of alkenes is the 1,3-dipolar cycloaddition reaction of ozone to the C=C double bond, which gives a 5-membered ring intermediate called a primary ozonide (POZ). This step is followed by a rapid decomposition reaction (see Fig. 1), in which the O–O and C–C bonds of POZ are cleaved together to yield a CI and a ketone product^20,21^.

**Figure 2:**
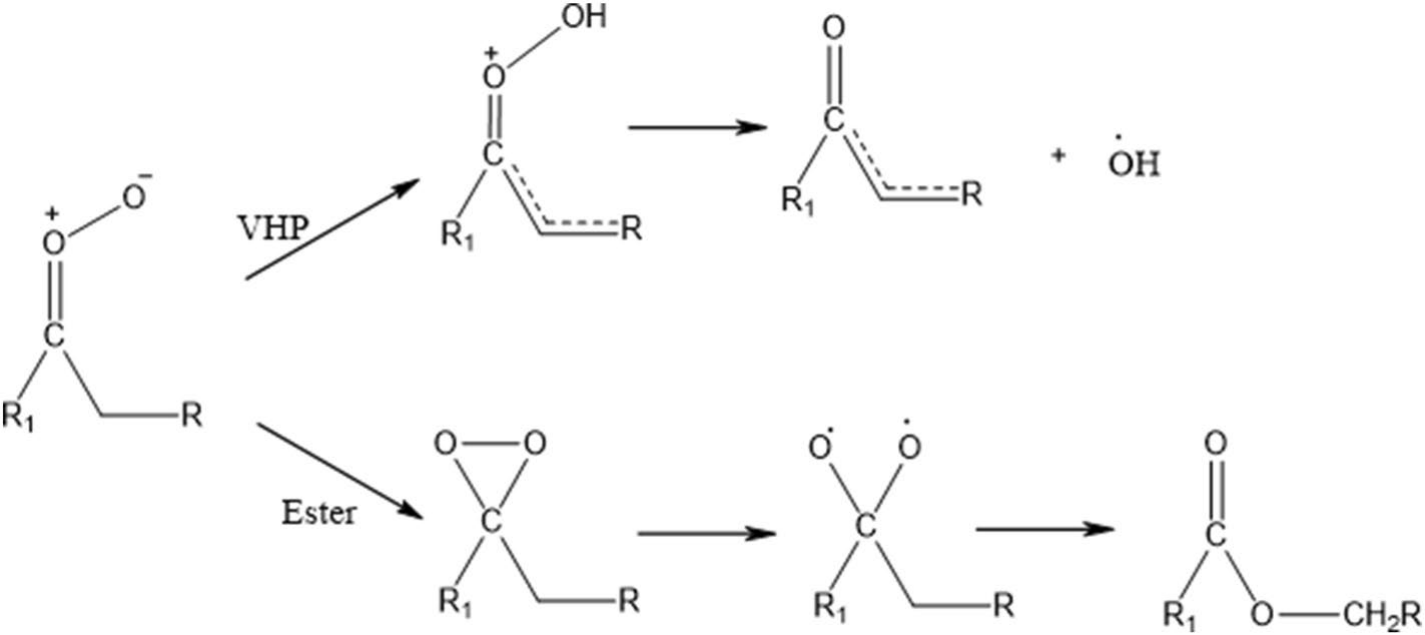
Proposed unimolecular (VHP and Ester) mechanistic pathways of the CI. ‘R1’ is a cyclic group.

The CI can undergo either a unimolecular^6^ or a bimolecular reaction^22^. More recently, Almatarneh et al.^23^ studied 16 pathways for the unimolecular and bimolecular decomposition of propylamine, which gave us a good glance about the performance of some levels of theory were used. The unimolecular reaction is known to form OH radicals through two possible pathways. The first pathway involves the isomerization of the CI to vinyl hydroperoxide (VHP) via a 1,4-proton shift in the carbonyl oxide, in addition to a breakage of the O–O bond either in a concerted step or through a VHP intermediate in the *syn*-CI conformation (see Fig. 2).

In the second pathway, a dioxirane intermediate is first formed via a cyclization reaction of the *anti*-CI conformation. Then the O–O bond cleaves to form a singlet biradical structure that leads to the formation of an ester (or an acid) through rearrangement, depending on the structure of substituent. Both pathways show a high dependency on the CI configuration (*syn*-CI or *anti-*CI conformations, see Fig. S1 in the supporting information (SI)).

In bimolecular reactions, the stabilized Criegee intermediates (sCIs) can react with atmospheric species, such as NO_2_, SO_2_^24–27^, OH, and water^22,28,29^. The branching of unimolecular-to-bimolecular reaction pathways can be acquired based on the relative magnitudes of CI energies in comparison with the energy barrier for *syn*-CI to *anti*-CI conformations and relevant concentrations of species in the competing reaction pathways^30^. The bimolecular reactions involving CIs, are inevitably to be the key processes in the formation of aerosols in the atmosphere. Furthermore, CI can act as an oxidant by transferring an oxygen atom to NO^27,31^ or CO, resulting in the formation of a carbonyl compound (aldehyde or ketone depending on the type of CI) and NO_2_ or CO_2_^28^. The oxidation of SO_2_ by CIs is an important source of sulfuric acid, which plays an important role in aerosol nucleation^22^. On the other hand, addition reactions can take place between atmospheric species that contain hydrogen atoms and CIs, along with the hydrogen transfer from small molecules to the terminal oxygen atom of CIs^32^. Kumar et al.^33^ studied the reactions of H–X (X = H, CH_3_, CH_2_F, CHF_2_, CF_3_, and SiH_3_) with the Criegee intermediate. They found that the reactions of H_2_ or SiH_4_ with CH_2_OO significantly have lower barriers than those for the CH_4_. They reported that the reaction of CH_2_OO–H_2_ is 9–11 orders of magnitude faster than the reaction of CH_2_OO–CH_4_ over the 200–300 K. Most importantly, they showed that the Criegee intermediate could be an interesting metal-free system that can activate small molecules (such as hydrogen, methane, silane).

Based on the above discussions, it is of great importance to understand the impact of methane in connection with CIs on the atmosphere and climate change. To our best knowledge, there has been only one computational study on the reaction of CI with methane in the literature. Based on DTF analysis, Xu et al. reported two reaction mechanisms of the sCIs with methane and different alkanes, which result in the formation of alcohol and hydroperoxide species^7^.

**Figure 3:**
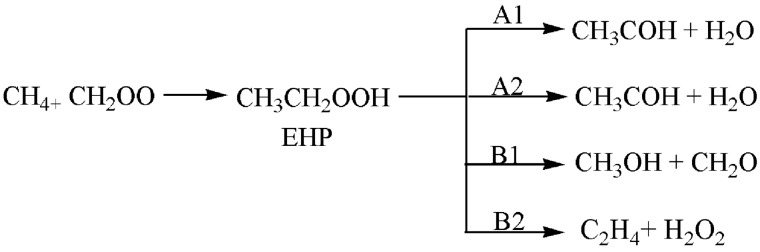
Proposed pathways for the unimolecular dissociation of EHP.

**Figure 4:**
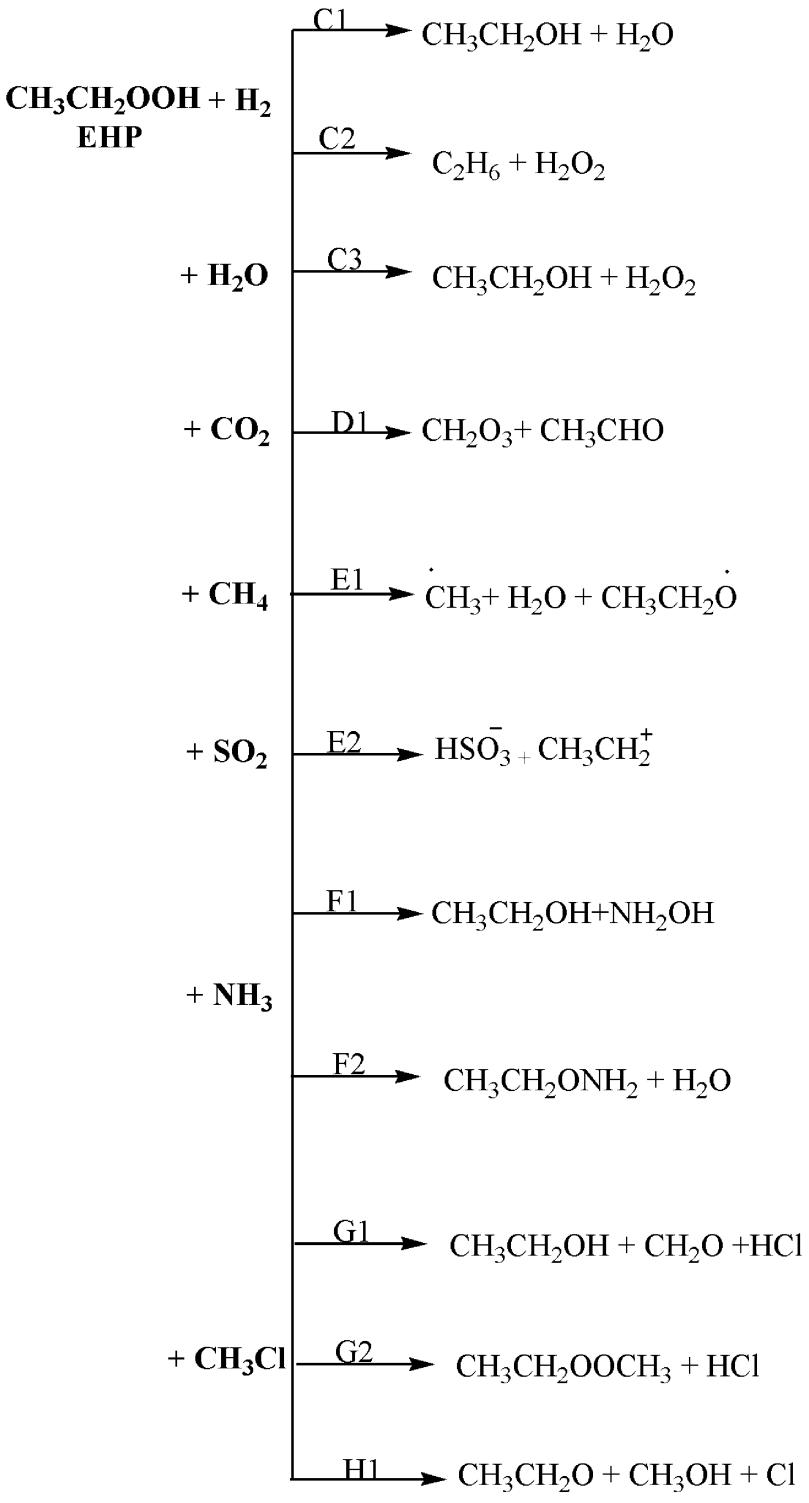
Proposed pathways for the bimolecular reactions of the CI with CH_4_.

In this work, a detailed computational study was conducted in order to locate the most plausible reaction mechanism. The four possibilities of the unimolecular dissociation of (CH_3_CH_2_OOH) were investigated. Furthermore, the bimolecular reactions of ethyl hydroperoxide with different gases were examined. Figures 3 and 4 show the proposed pathways for unimolecular dissociation and bimolecular reactions of ethyl hydroperoxide (EHP). Thermodynamic and kinetic parameters were obtained at different levels of theory. The major goals are to help in the clarification of the reaction mechanisms, to identify the most plausible reaction routes, and to understand the chemistry of bimolecular reactions. It is envisioned that this computational study would be of interest to experimentalists by providing more detailed information about this reaction and can possibly aid in the design of new experiments for the development of useful synthetic methods.

## Computational methods

All calculations were performed utilizing the Gaussian 09 (G09) software package^34^. Transition states (TS) were confirmed by having one imaginary frequency along the designated reaction pathway, while minimized geometries of reactant, intermediates and products were validated as minima with no imaginary frequencies. The optimization was performed utilizing Becke’s three-parameter hybrid method using the LYP correlation functional (B3LYP)^35,36^, and global Minnesota hybrid functional (M06-2X)^37^. The B3LYP geometries were optimized using the 6-31G(d), 6-31G (2df,p), and 6-311++G(3df,3pd) basis sets. The B3LYP/6-31G(d) geometries were performed utilizing amplitude probability frequency distribution (APFD)^38^, and the Minnesota 11 functional (M11) was created from optimized a new range separated hybrid meta-GGA (generalized gradient approximation) functional correct to second order in both the exchange and the correlation parts^39^. The impact of solvent (in our study is water) on the reaction mechanism was evaluated. The geometries for all pathways were fully optimized using the polarizable continuum solvation model (PCM)^40^ and the solvation model based on density (SMD)^41^. The optimized structures using PCM and SMD calculations were performed at the B3LYP/6-311++G(3df,3pd) level of theory. Relative energies of all stationary points are corrected with zero-point vibrational energies (ZPE). Furthermore, transition states were evaluated using the intrinsic reaction coordinate (IRC)^42^ method at the B3LYP/6-31G(d) level of theory to a certain the connection of the reactants and products/intermediates on the potential energy diagrams (PEDs).

## Results and discussion

In this study, comprehensive quantum chemistry calculations of fifteen possible reaction pathways were conducted for unimolecular dissociation and bimolecular reactions of EHP. Pathways **A1**, **A2**, **B1**, and **B2** are unimolecular dissociation of (CH_3_CH_2_OOH), while pathways **C1**, **C2**, **C3**, **D1**, **E1**, **E2**, **F1**, **F2**, **G1**, **G2**, and **H1** are bimolecular reactions. The kinetic and thermodynamic parameters for all considered pathways were calculated at different levels of theory.

### The structure of the simplest Criegee intermediate and methane

**Figure 5:**
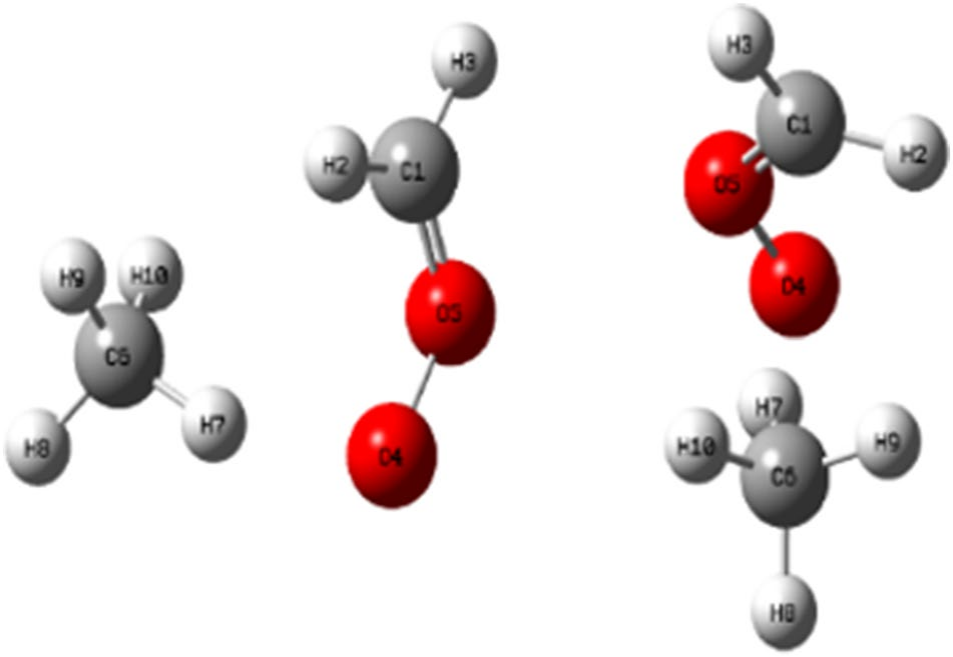
The optimized geometry of sCIs and methane (side and front views), obtained at the B3LYP/6-311++G(3df,3pd).

The structure of the simplest Criegee intermediate with methane has been optimized using the B3LYP/6-311++G(3df,3pd) method. Figure 5 depicts the optimized ground state geometry. The selected optimized structural parameters for reactant using B3LYP/6-311++G(3df,3pd) method, are listed in Table SI.

### The potential energy diagram (PED) for Criegee intermediate reaction with methane, pathways A1, A2, B1, and B2

There are four possible pathways for the reaction of CI with methane, which are referred to as Pathways **A1**, **A2**, **B1** and **B2**. These pathways involve a two-step mechanism. The first step is the formation of an intermediate as an initial pre-reactive complex by an intermolecular H-bond between the terminal oxygen atom of CH_2_OO and one of the hydrogen atoms of methane.

The second step is a concerted dissociation of the intermediate into different products. In pathway **A1**, it was found that both CH_2_OO and CH_4_ are close to each other and form an intermolecular H-bond between them. There is a slight increase in the O–O bond of CH_2_OO, with the C–H bond involved in the H-bond elongated. After that, the H and CH_3_ fragments are coupled with the terminal O and C atoms of CH_2_OO, resulting in the formation of the ethyl hydroperoxide (EHP). In TS1A1 a large geometrical change can be observed. For instance, the O–O bond is extended by about 0.04 Å. Meantime, the C–H bond of CH_4_ involved in the H-bond is extended by 0.219 Å. In contrast, the C atoms of the CH_2_OO and CH_4_ approach each other, with the distance between two C atoms decreased to 1.566 Å. Similarly, the H-bond distance is decreased to 1.273 Å.

**Figure 6:**
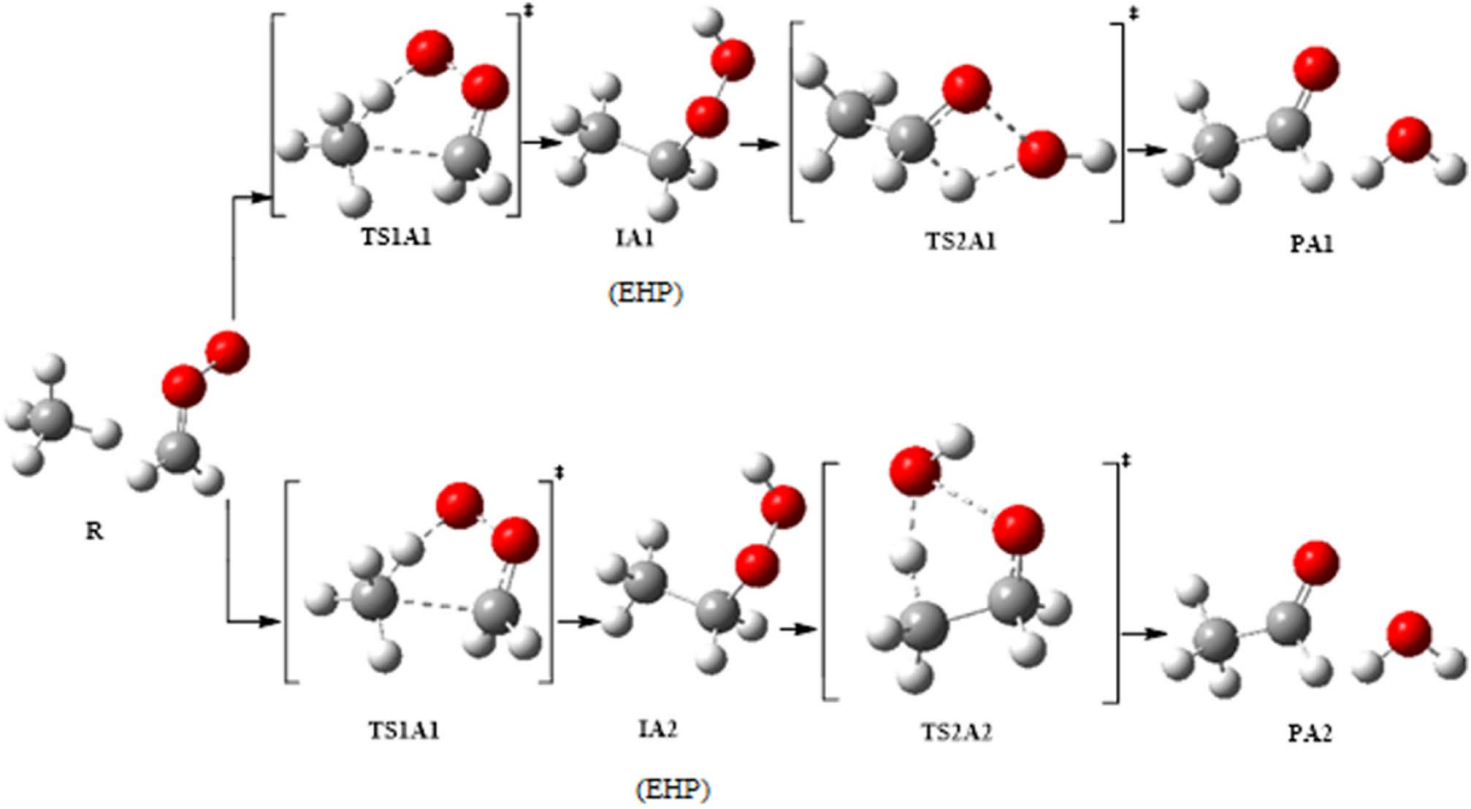
Optimized geometries for Criegee intermediate reaction with methane at B3LYP/6-311++G(3df,3pd). Pathways **A1** and **A2**.

Ethyl hydroperoxide is an intermediate in the second step of reaction with an intermolecular H-bond formed between the terminal oxygen atom of (CH_3_CH_2_OOH) and one of the hydrogen atoms in the molecule. This step then leads to the formation of ethanal and water. The optimized structures of all stationary points for pathway **A1** are shown in Fig. 6. There are two reaction pathways to form CH_3_CHO and H_2_O through TS2A1 and TS2A2. Between them, the pathway with a four-member-ring transition state (TS2A1) is dominant. In pathway A2, H_2_O is eliminated from CH_3_CH_2_OOH via a five-member-ring transition state (TS2A2). Different from pathway **A1**, the H atom of the eliminated H_2_O in pathway A2 originates from the C6 atom as illustrated in Fig. 6.

**Figure 7:**
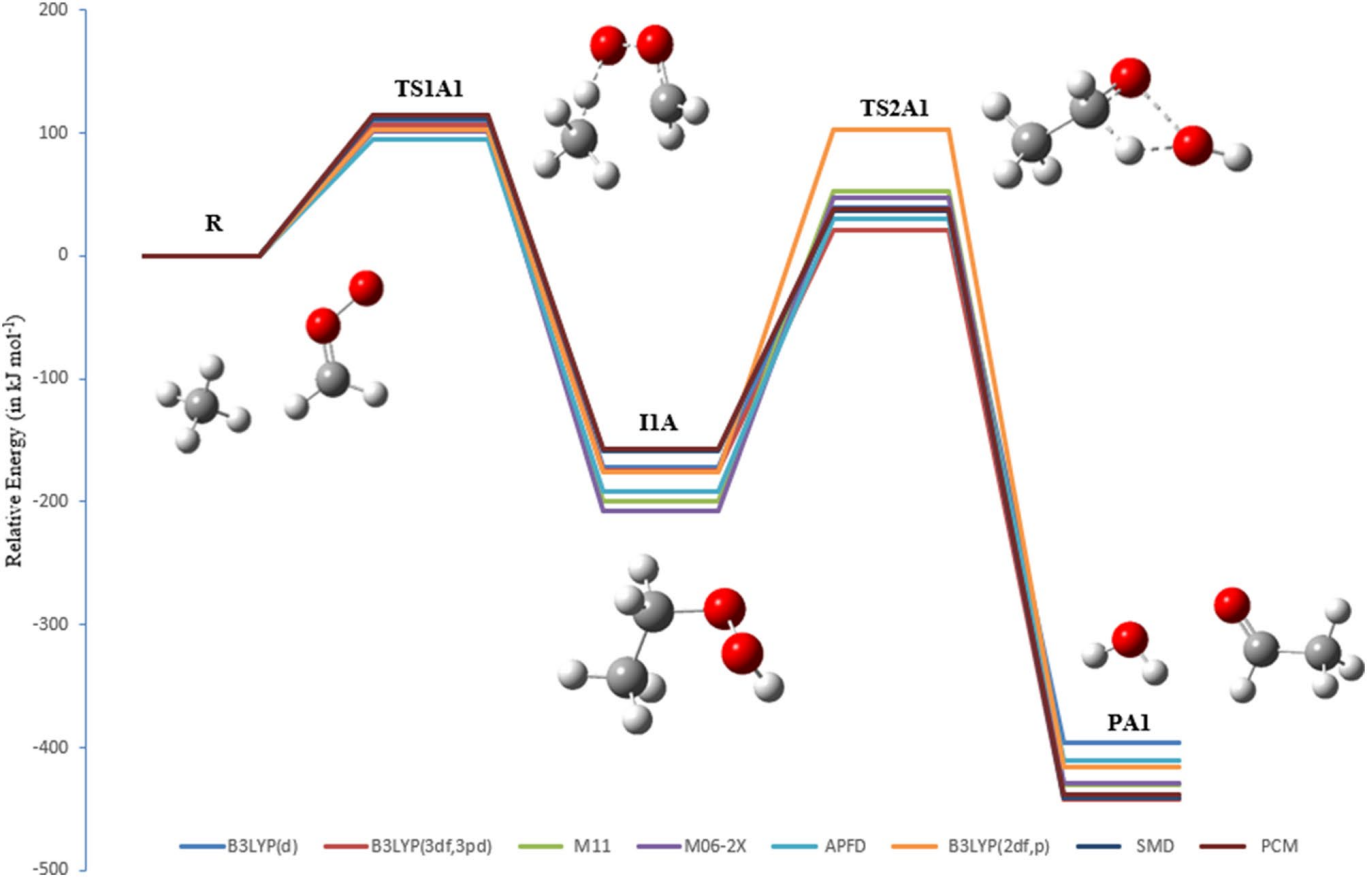
PED for the reaction of EHP with H_2_O (Pathway **A1**). Relative energies for several calculation methods are given in kJ mol^−1^.

**Figure 8:**
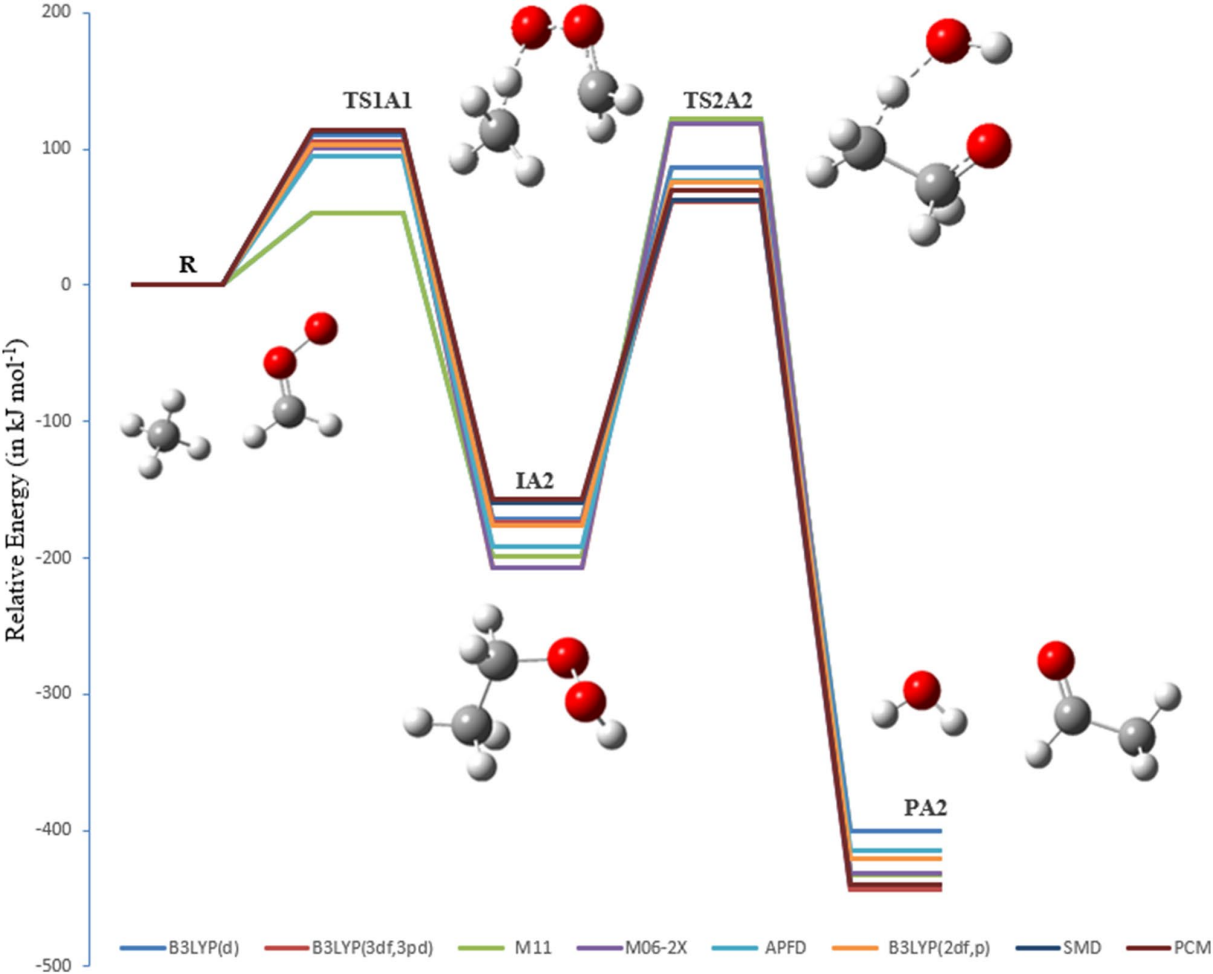
The PED for the reaction of CI with CH_4_ (Pathways **A2**). Energies calculated at several levels of theory.

The PEDs at different methods for pathways **A1** and **A2** are shown in Figs. 7 and 8. The activation energies of TS2A1 and TS2A2 at the B3LYP/6-311++G(3df,3pd) level of theory are 196 and 235 kJ mol^−1^, respectively. Furthermore, the respective activation energies at B3LYP/6-31G(d) are 210 and 258 kJ mol^−1^ for TS2A1 and TS2A2 (see Table 1). By comparison, the activation energy of TS2A2 is higher than that of TS2A1. Therefore, pathway A1 is more favored than pathway A2. However, with the solvation effect taken into account, there is a little change in the activation energy for the first step. Employment of PCM and SMD models for the second step did not result in significant changes in the energy barrier for TS2A1, but the barrier for TS2A2 is decreased to 227 (PCM) and 222 kJ mol^−1^ (SMD) at the B3LYP/6-311++G(3df,3pd) level of theory. Values of Gibbs reaction energies call for the attention of the entropic contribution.

**Table 1:** Activation energies and Gibbs energies of activation for the Reaction of CI with methane (in kJ mol^−1^) at 298.15 K (Pathways **A1** and **A2**).

Theory/basis set	TS1A		TS2A1		TS2A2	
	E_a_	ΔG^‡^	E_a_	ΔG^‡^	E_a_	ΔG^‡^
B3LYP/6-31G(d)	111	132	210	210	258	259
B3LYP/6-311++G(3df,3pd)	106	131	196	195	235	235
M11/6-31G(d)	101	115	252	252	322	324
M06-2X/6-31G(d)	101	117	255	254	326	327
APFD/6-31G(d)	94	113	223	222	269	270
B3LYP/6-31G(*2*df,p)	103	125	279	280	251	253
SMD	112	139	196	196	222	205
PCM	114	153	195	194	227	227

**Figure 9:**
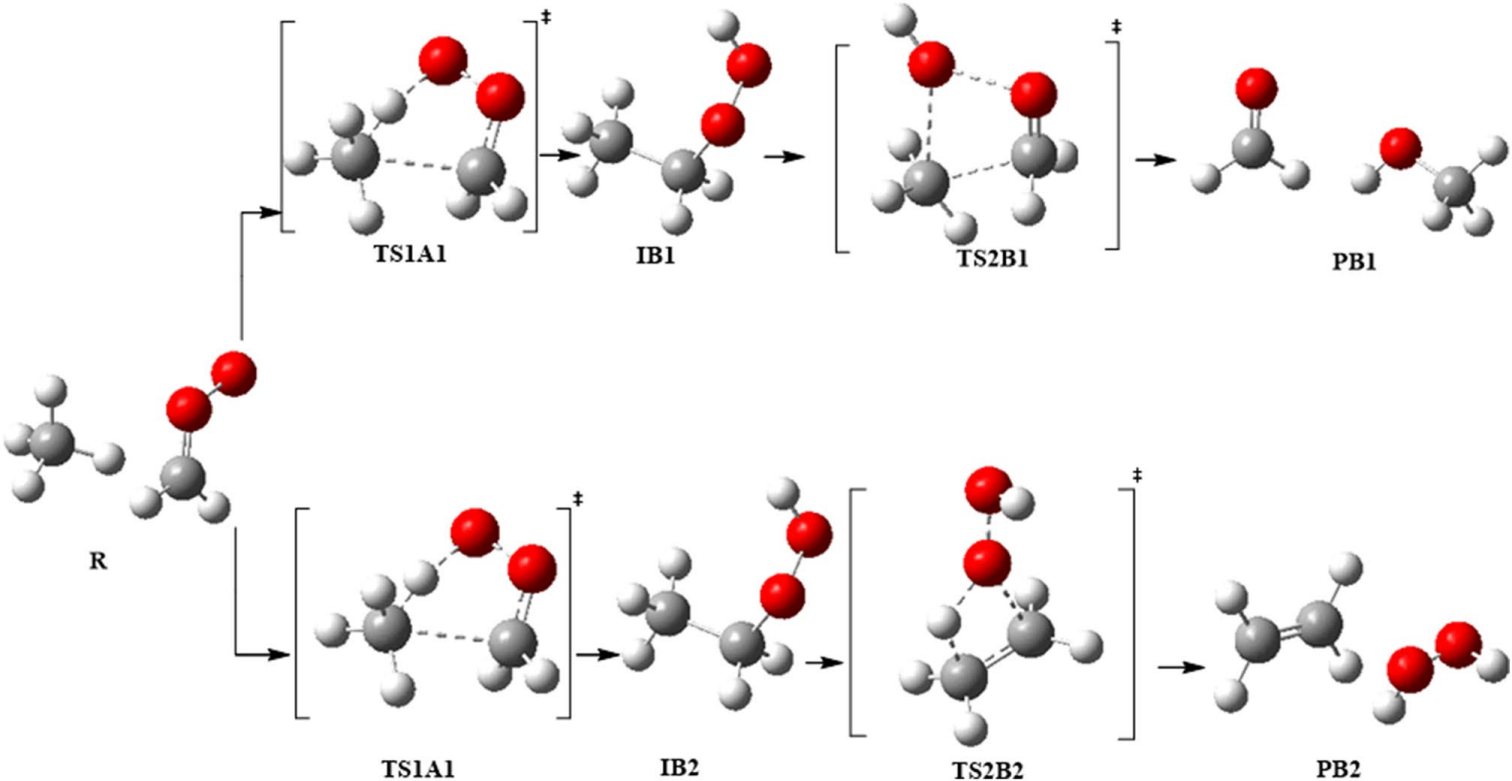
Optimized geometries for Criegee intermediate reaction with methane at the B3LYP/6-311++G(3df,3pd), Pathways **B1** and **B2**.

The formation of methanol and formaldehyde occurs through the transition state TS2B1as shown in Fig. 9. In pathway **B1**, the terminal O4 atom of CH_3_CH_2_OOH gradually approaches the C6 atom, the O4–O5 and C1–C6 bonds mentioned above are further extended. As a result, the strength of the O4–O5 and C1–H6 bonds are extremely weakened. Conversely, the interaction of O4⋯C6 bond is strengthened. The distance between O4–O5 bond is increased by 0.519 Å, and the double bond is formed between C1 and O5 with a bond distance of 1.252 Å. The C1–C6 bond is broken and the distance is 2.173 Å. Finally, methanol is formed by decreasing the distance between C6–O4 to 1.495 Å.

In TS1B1 the calculated activation energies are 106 and 94 kJ mol^−1^ at B3LYP/6-311++G(3df,3pd) and APFD/6-31G(d) levels of theory, respectively. However, lower activation energies are obtained at the APFD/6-31G(d) level of theory (see Table 2). The energy value at the APFD/6-31G(d) is in good agreement with the value of 90 kJ mol^−1^ obtained for the activation of methane by oxygen and nitrogen oxide^9^.

**Table 2:** Activation energies and Gibbs energies of activation for the Reaction of CI with methane (in kJ mol^−1^) at 298.15 K (Pathways **B1** and **B2**).

Theory/basis set	TS1A		TS2A1		TS2A2	
	E_a_	ΔG^‡^	E_a_	ΔG^‡^	E_a_	ΔG^‡^
B3LYP/6-31G(d)	111	132	255	254	255	253
B3LYP/6-311++G(3df,3pd)	106	131	234	234	234	234
M11/6-31G(d)	101	115	396	395	284	284
M06-2X/6-31G(d)	101	117	289	289	289	289
APFD/6-31G(d)	94	113	366	366	264	264
B3LYP/6-31G(*2*df,p)	103	125	337	336	244	245
SMD	112	139	219	215	226	224
PCM	114	153	230	227	228	228

The respective activation energies for the rate-determining steps (TS2A1 and TS2B1) at the B3LYP/6-311++G(3df,3pd) level of theory are 196 and 234 kJ mol^−1^, respectively. Moreover, the activation energies of TS2A1 and TS2B1 at the B3LYP/6-31G(d) level of theory are 210 and 255 kJ mol^−1^, respectively. By comparison, the activation energy of TS2B1 at B3LYP/6-31G(d) level of theory is lower by 28 kJ mol^−1^ than the value obtained at α-methoxy hydroperoxide decomposition energy for the addition of methanol to the CI^11^. The energy value of TS2B1 at the M06-2X/6-31G(d) level of theory is lower by 46 kJ mol^−1^ than the value obtained from the ozonolysis of phenanthrene study^11^ at the same level of theory. Furthermore, the water phase reduces the energy barrier of TS2B1 to 230 and 219 kJ mol^−1^ using PCM and SMD solvation models, respectively, at B3LYP/6-311++G(3df,3pd) level of theory.

In the reaction mechanism of pathway **B2**, H_2_O_2_ eliminates from EHP via a planar four-member ring transition state TS2B2. The distance between C1 and O5 is increased to 1.933 Å, and the double bond formed between C1–C6 is 1.399 Å in length, and the C6-H8 bond is to be broken after the transition state. Finally, the distance between H8–O5 is decreased to 1.819 Å, and then hydrogen peroxide and ethylene are formed (see Fig. 11).

**Figure 10:**
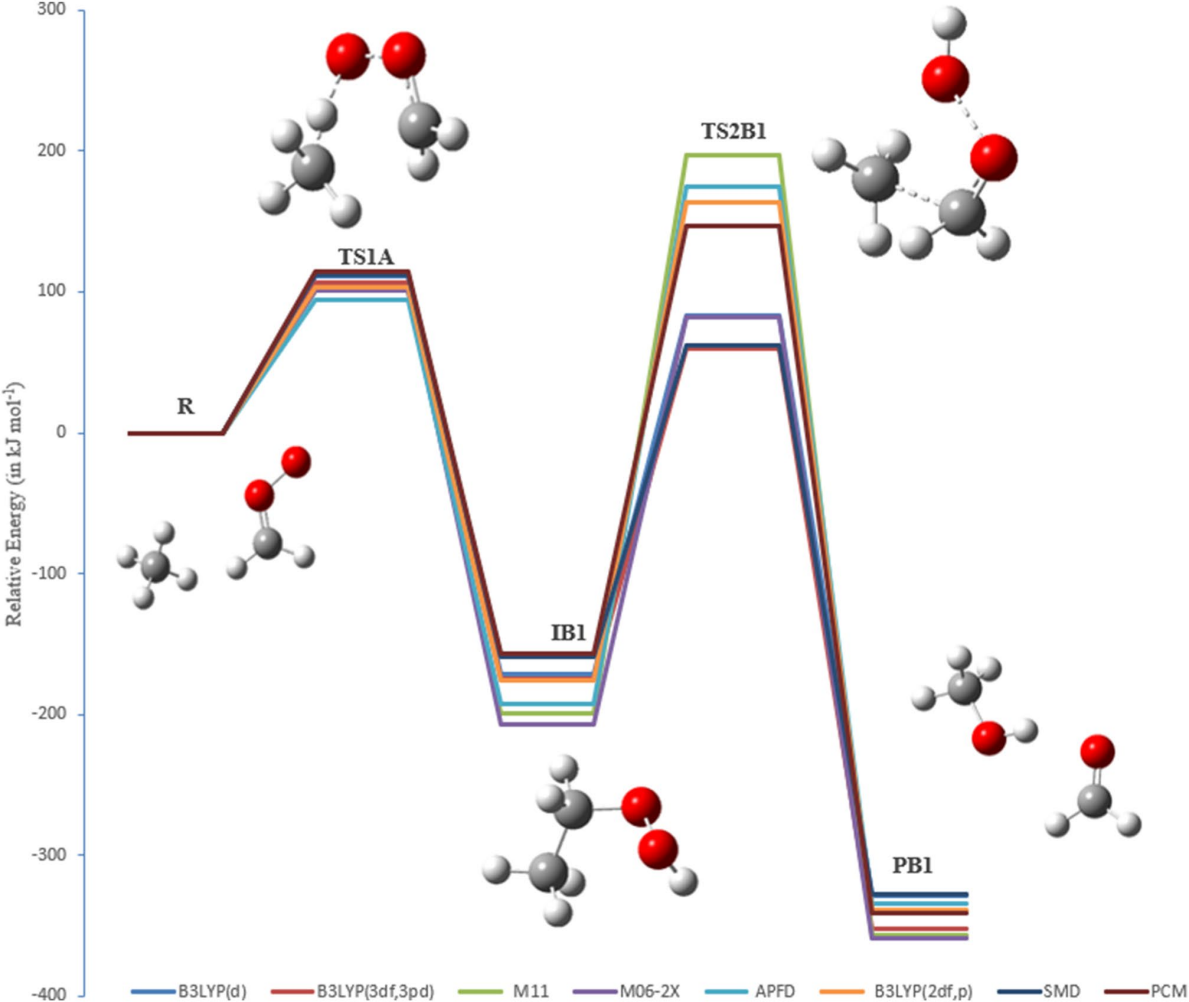
The PED for the reaction of CI with CH_4_ (Pathway **B1**). Energies calculated at several methods.

**Figure 11:**
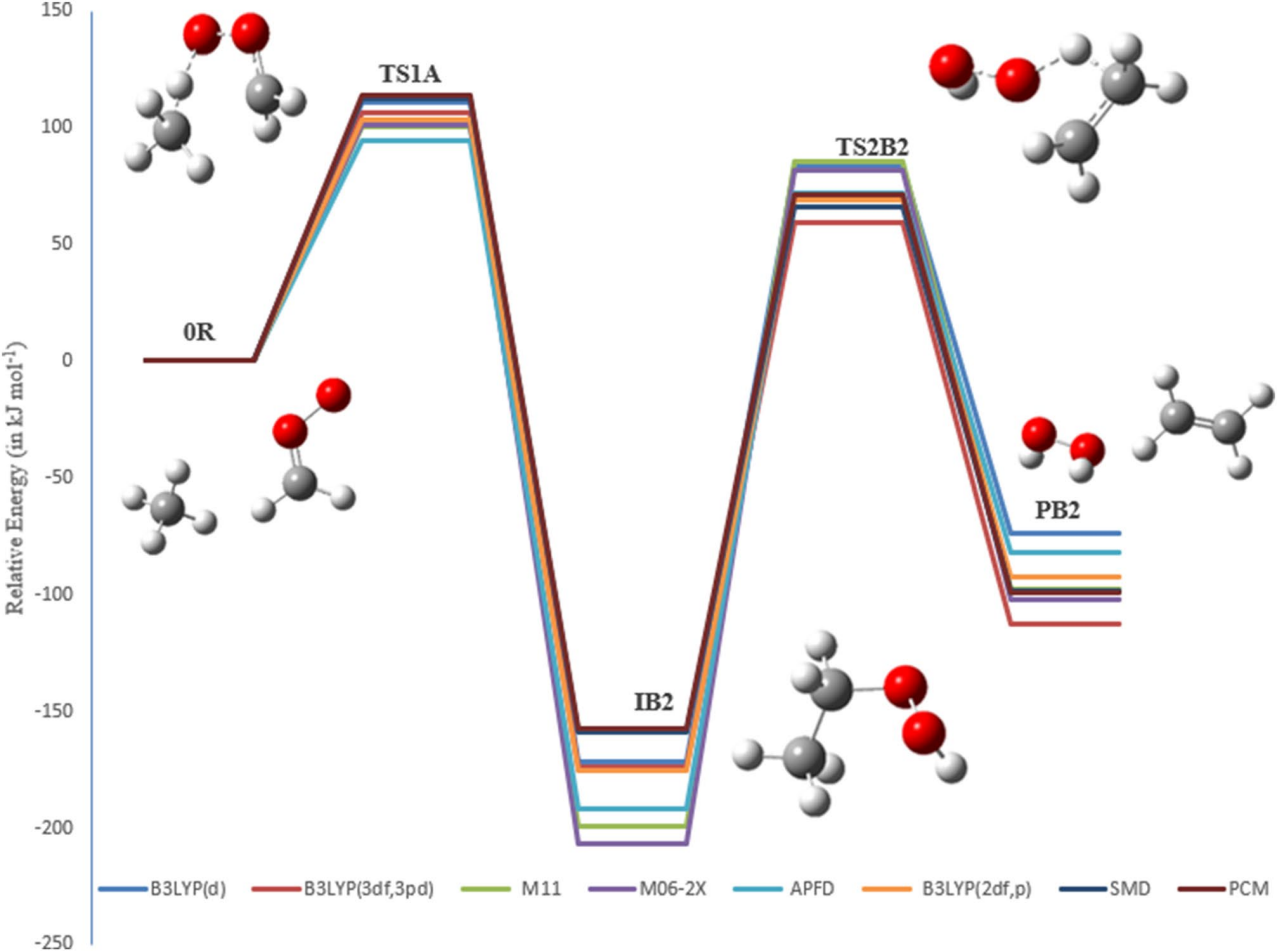
The PED for the reaction of CI with CH_4_ (Pathway **B2**). Energies calculated at several methods.

However, all bond lengths in TS2A1, TS2A2, TS2B1 and TS2B2 are in excellent agreement with the reported values for the unimolecular decomposition of ethyl hydroperoxide^43^. The thermodynamic properties of these pathways were found to be exothermic and exergonic at all methods. This indicates that the reaction favors the forward direction. The optimized structures for pathways **B1** and **B2** are included on the PEDs using different methods are depicted in Figs. 10 and 11.

### The reaction of EHP with H_2_ and H_2_O molecules (pathways C1 → C3)

**Figure 12:**
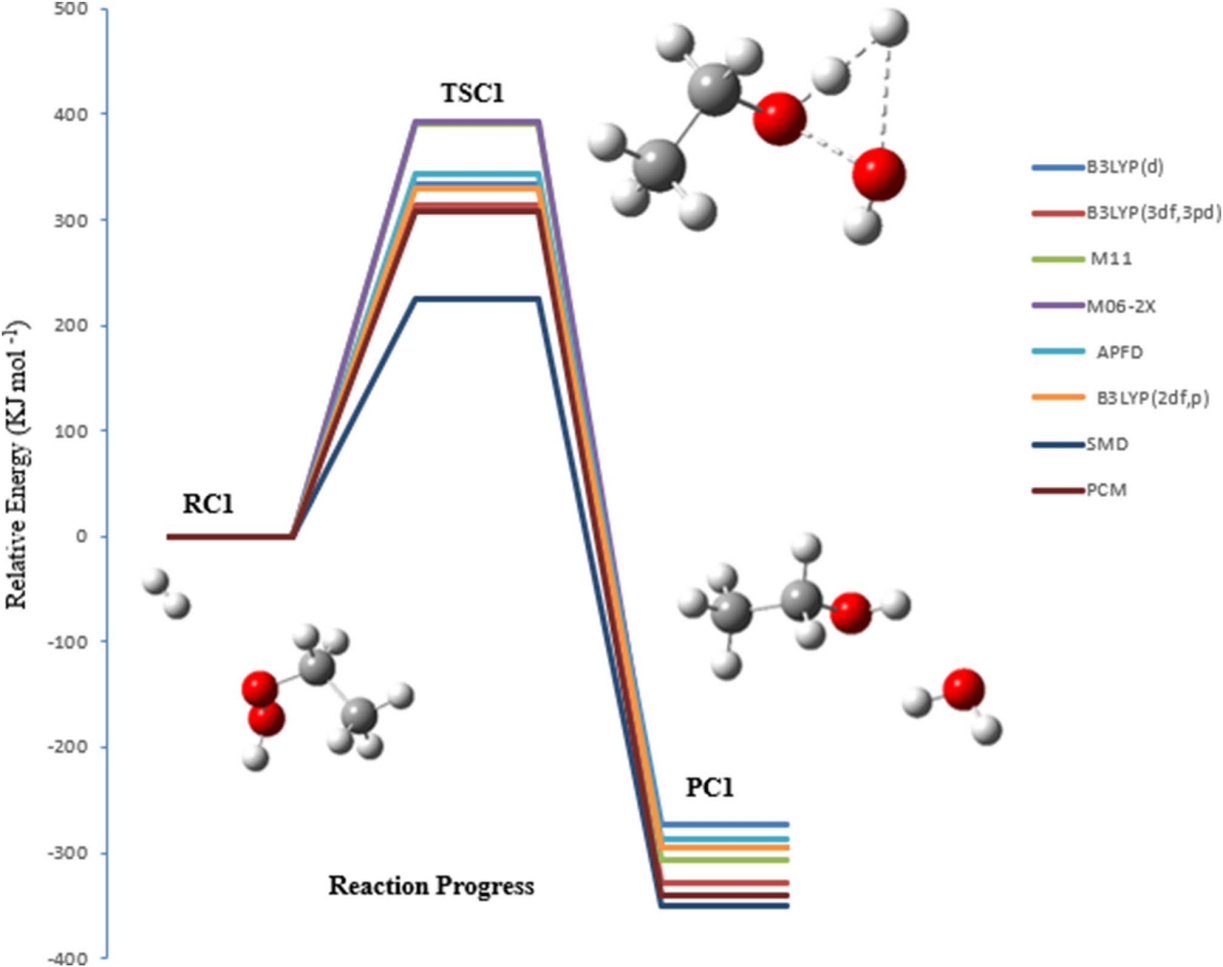
PED for the reaction of EHP with H_2_ (pathway C1). Relative energies at several methods are given in kJ mol^−1^.

**Figure 13:**
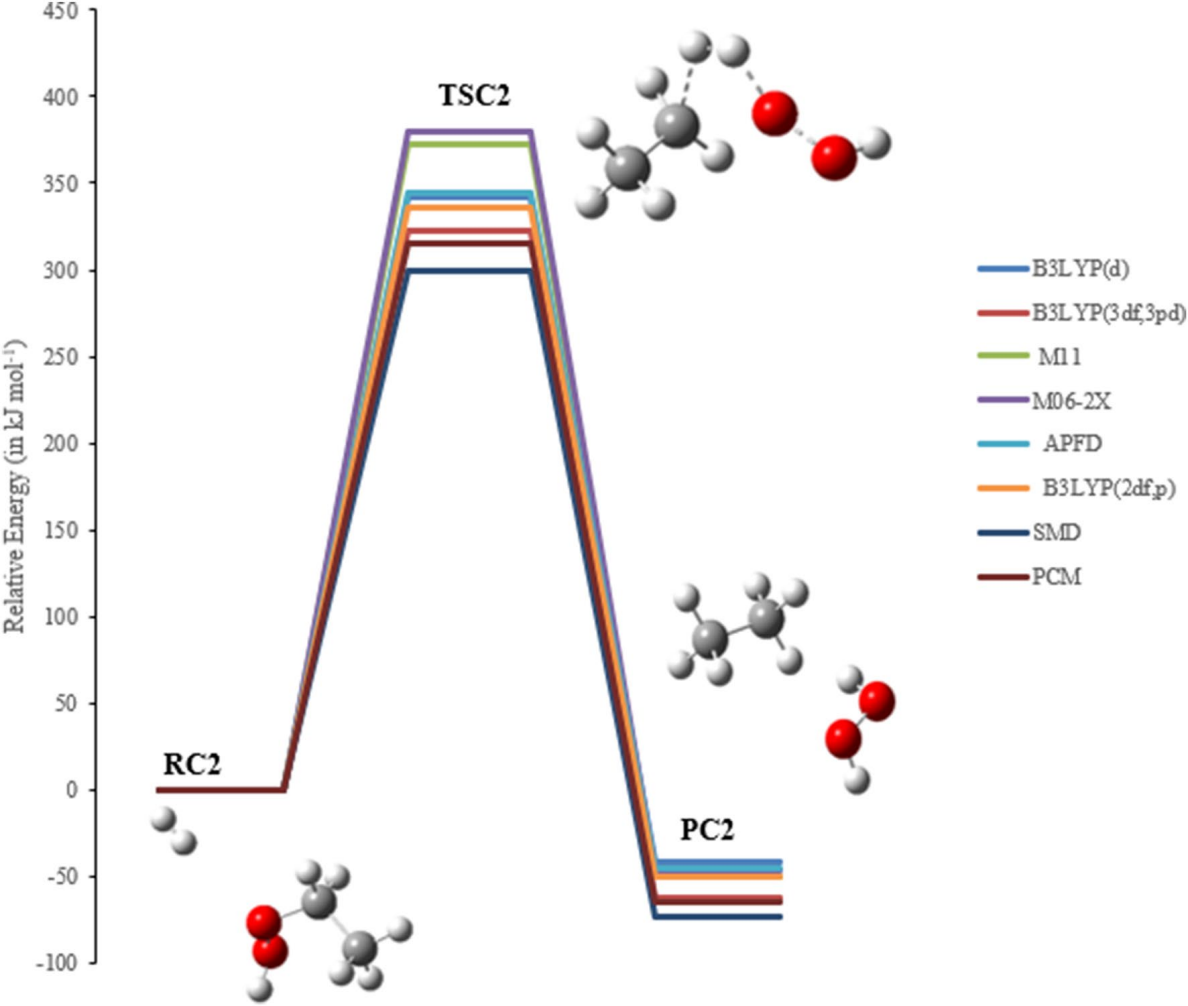
PED for the reaction of EHP with H_2_ (pathway C2). Relative energies at several methods are given in kJ mol^−1^.

**Figure 14:**
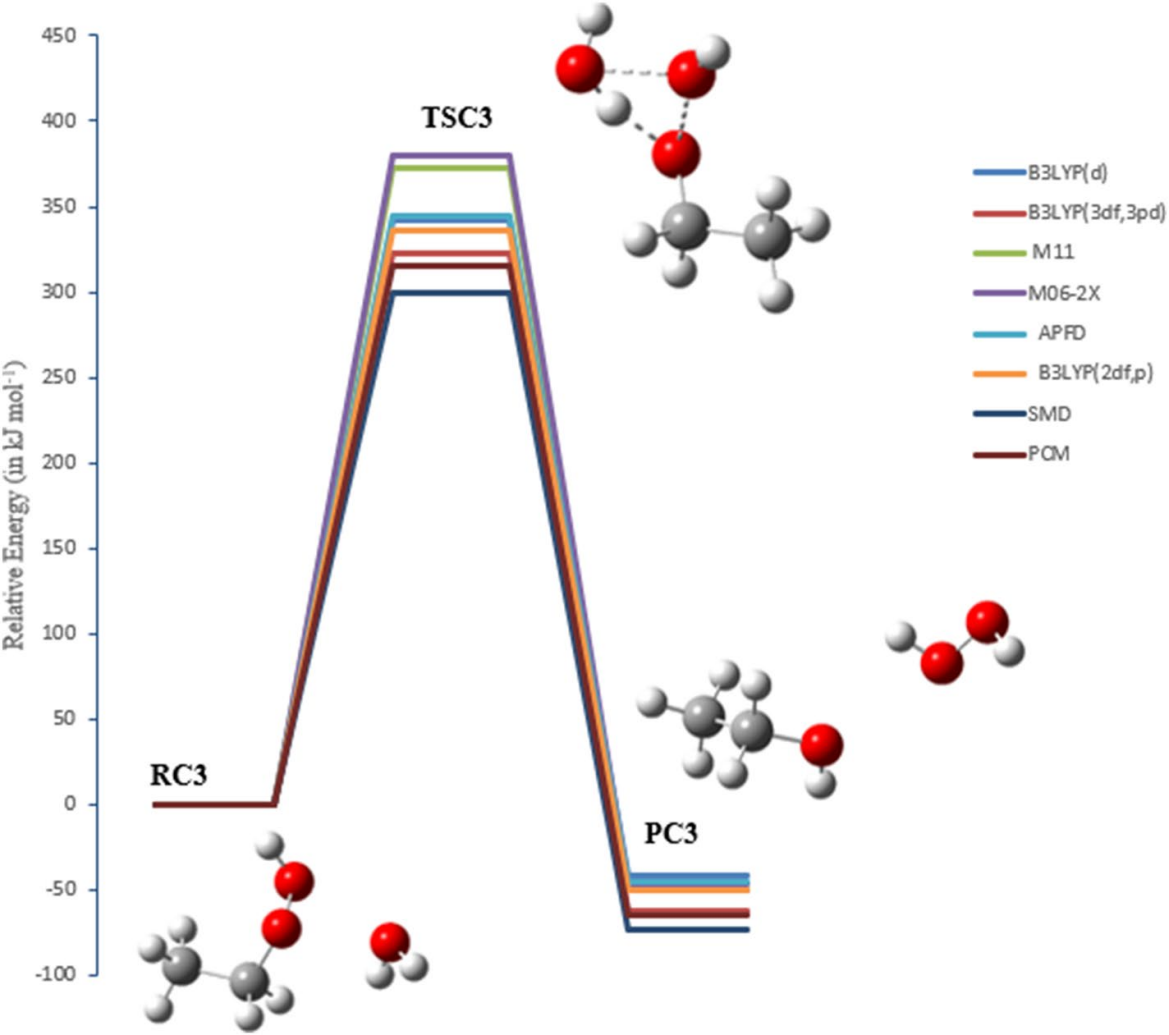
PED for the reaction of EHP with H_2_O (pathway C3). Relative energies at several methods are given in kJ mol^−1^.

Two pathways were investigated for the bimolecular reaction of ethyl hydroperoxide with hydrogen, which are denoted as pathways **C1** and **C2**. Pathway **C3** is with a water molecule. Figures 12, 13 and 14 illustrate the equilibrium geometries of the reactants, transition states, and products that are included on the PED for pathways **C1**, **C2** and **C3**. The activation energies and Gibbs energies of activation for the reaction are listed in Table 3.

**Table 3:** Activation energies and Gibbs energies of activation for the Reaction of CI with methane (in kJ mol^−1^) at 298.15 K (Pathways **C1 → C3**).

Theory/basis set	TSC1		TSC2		TSC3	
	E_a_	ΔG^‡^	E_a_	ΔG^‡^	E_a_	ΔG^‡^
B3LYP/6-31G(d)	333	347	342	354	313	320
B3LYP/6-311++G(3df,3pd)	313	329	323	340	393	302
M11/6-31G(d)	390	394	373	381	370	371
M06-2X/6-31G(d)	393	403	345	357	373	376
APFD/6-31G(d)	343	356	345	357	323	328
B3LYP/6-31G(*2*df,p)	329	341	355	346	317	326
SMD	292	310	300	310	422	427
PCM	307	323	315	329	339	343

In TSC1 and TSC2 the activation energies at the B3LYP/6-31G (2df,p) level of theory are compatible, with values of 329 and 355 kJ mol^−1^, respectively. Furthermore, the activation energies for TSC1 and TSC2 at the B3LYP/6-311++G(3df,3pd) level of theory are somewhat similar, with values of 313 and 323 kJ mol^−1^, respectively. The activation energy values for both pathways are in good agreement with the B3LYP/6-31G(d), M11/6-31G(d), APFD/6-31G(d), and M06-2X/6-31G(d) levels of theory. Hence, the reaction is more favorable at the B3LYP/6-311++G(3df,3pd) level of theory. Moreover, the effect of solvation using PCM and SMD models for pathway C1 and C2 reduces the overall activation energy to 307 and 315 kJ mol^−1^ at PCM, and to 292 and 300 kJ mol ^−1^ at SMD, respectively, at B3LYP/6-311++G(3df,3pd) level of theory. It should be noted here that the barriers using SMD solvation model are lower than the PCM calculated values for both pathways C1 and C2.

In general, the decomposition of EHP is disfavored to occur in the gas phase owing to sizable activation barriers. However, water plays an important role to make it more efficient. The reaction of the EHP with H_2_O, denoted as pathway **C3**, along with the effect of water on the barrier, were investigated. The purpose of exploring this pathway is to clarify the atmospheric fate of EHP within the aqueous phase. The reaction products formed during the photolysis and OH initiated oxidation of EHP under recreated atmospheric water conditions are CH_3_CH_2_OH and H_2_O as shown in the following reactions.

$$ \begin{array}{*{20}l} {{\text{CH}}_{{3}} {\text{CH}}_{{2}} {\text{OOH }} + {\text{ hv}} \to {\text{CH}}_{{3}} {\text{CH}}_{{2}} {\text{O}}^{ \cdot } +^{ \cdot } {\text{OH}}} \hfill \\ {{\text{CH}}_{{3}} {\text{CH}}_{{2}} {\text{O}}^{ \cdot } + {\text{ H}}_{{2}} {\text{O}} \to {\text{CH}}_{{3}} {\text{CH}}^{ \cdot } \left( {{\text{OH}}} \right) \, + {\text{ H}}_{{2}} {\text{O}}} \hfill \\ {{\text{H}}_{{2}} {\text{O }} + {\text{ hv}} \to^{ \cdot } {\text{OH }} +^{ \cdot } {\text{H}}} \hfill \\ {^{ \cdot } {\text{OH }} +^{ \cdot } {\text{OH}} \to {\text{H}}_{{2}} {\text{O}}_{{2}} } \hfill \\ {{\text{CH}}_{{3}} {\text{CH}}^{ \cdot } \left( {{\text{OH}}} \right) \, +^{ \cdot } {\text{H}} \to {\text{CH}}_{{3}} {\text{CH}}_{{2}} {\text{OH}}} \hfill \\ \end{array} $$

The bond length of O–OH (in EHP) increases from 1.463 to 2.356 Å. Followed this is an elongation of 0.094 Å in the other hydrogen bond that is being transferred to the central O atom to generate ethanol. The distance between OH–OH groups is decreased to 1.02 Å and then H_2_O_2_ is formed (see Fig. 14). The activation energy of TSC3 at the M06-2X/6-31G(d) levels of theory is in good agreement with M11/6-31G(d), with energy value being 373 kJ mol^−1^. The lowest energy barrier is obtained at the B3LYP/6-31G(d) level of theory. This energy barrier value is in good agreement with the B3LYP/6-31G (2df,p). The higher energy value is calculated at the B3LYP/6-311++G(3df,3pd). It should be noted that there is a significant solvent effect in pathway **C3**, which increases the energy barrier. Several sites are available for strong solute–solvent hydrogen-bonding interactions.

### The bimolecular reaction of the ethyl hydroperoxide with CO_2_, CH_4_ and SO_2_ gases (pathways D1, E1, E2)

**Figure 15:**
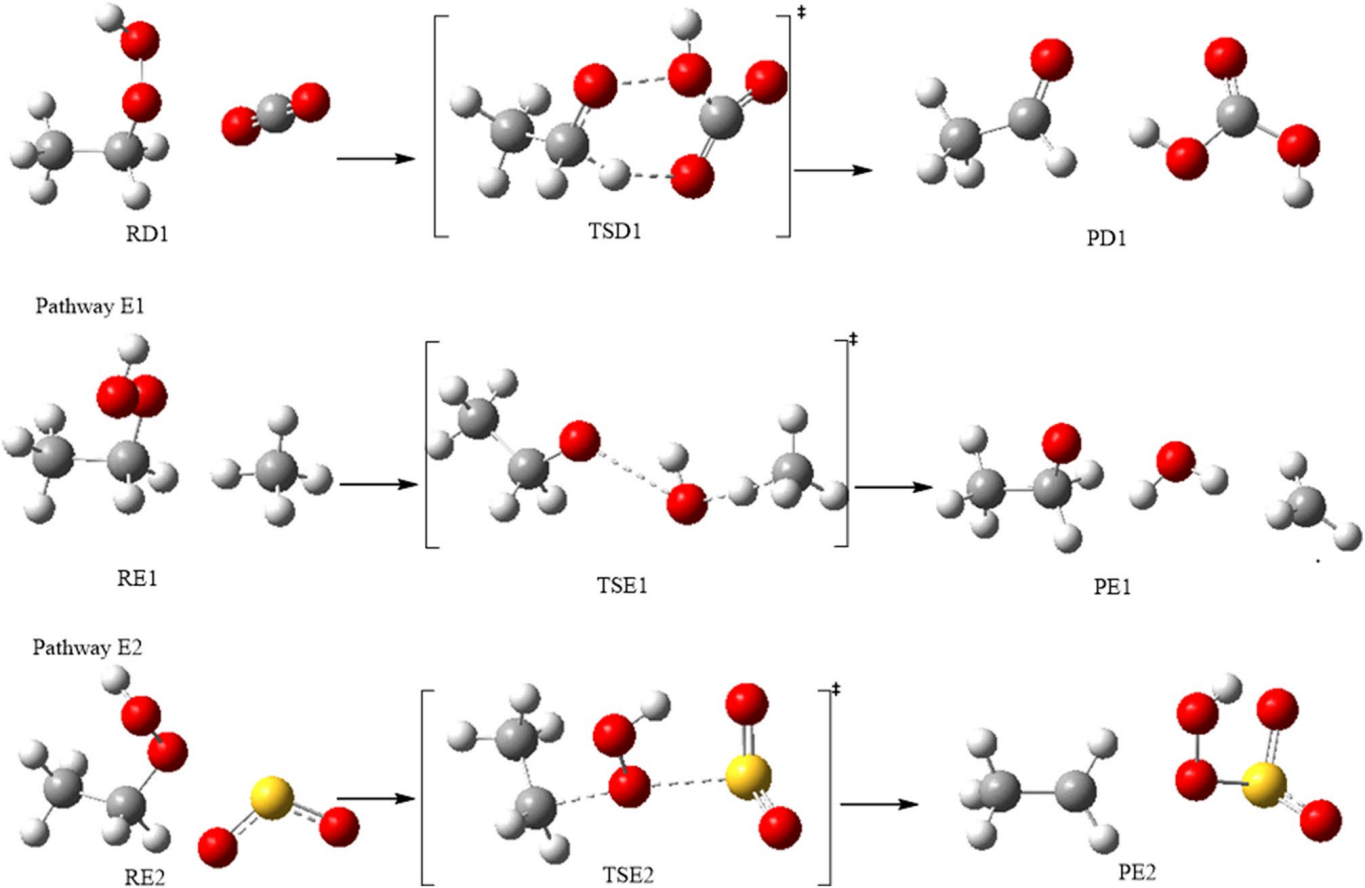
Optimized geometries for EHP with CO_2_, CH_4_, SO_2_ gases at the B3LYP/6-311++G(3df,3pd). Pathways (D1, E1, E2).

The reactions of EHP with carbon dioxide, methane and sulfur dioxide were studied computationally. Figure 15 shows the reaction mechanism for pathways **D1**, **E1**, and **E2**. Carbonic acid, formed by the hydration of carbon dioxide, assumes a significant role in numerous fields. It has scarcely received any consideration since it is believed to quickly disintegrate into carbon dioxide and water. Nonetheless, it has been found that without water vaporous carbonic acid is incredibly stable. For pathway **D1**, H_2_O results from EHP and reacts with CO_2_ via transition state TSD1, with an extensive change in the main bond lengths. Particularly, the decomposition of EHP occurs in a concerted step through dissociation of the O–OH group, where the bond is elongated from the molecule by 0.40 Å to become 1.83 Å, and OH is close to the carbon atom in CO_2_. Moreover, a double bond between C1 and O5 atoms is formed with a distance of 1.334 Å. The C1–H3 bond is further elongated to 1.156 Å, and H_2_O is then eliminated. After that, H3 is attached to the C atom in CO_2_ molecule, leading to C=O bond cleavage and the formation of carbonic acid and acetaldehyde (PD1). It should be noted that the C=O, O–O, and C–O bond lengths are 1.222 Å, 1.453 Å, and 1.366 Å at all levels of theory. This is consistent with the reported geometries in the literature^44^.

Pathway **E1** represents the reaction of the EHP with CH_4_, along with the impact of photochemical reactions on the barrier. Chemical reactions that include peroxyl and alkyl radicals have a significant influence on the atmospheric degradation of organic materials. Most alkyl and peroxyl radicals are generally unstable. Consequently, it is challenging to monitor their fates through experimental methods. Quantum chemical calculations have become an increasingly useful tool for the determination of gas-phase thermochemistry. Alkyl radicals are important intermediates in oxidation, pyrolysis, and photochemical reactions of hydrocarbons.

The mechanism of methane addition to ethyl hydroperoxide is investigated through TSE1. In the first step of the radical chain mechanism for the reaction of methane with EHP (initiation), ultraviolet light causes the weak EHP bond to undergo homolytic cleavage to generate ethoxy and hydroxyl radicals and starts the chain process. In the propagation step, the hydroxyl radical abstracts methane hydrogen to form H_2_O and a methyl radical. Finally, in the termination step, various reactions between the possible pairs of radicals allow for the formation of ethyl methyl ether. This step removes radicals and terminates the propagation cycle.

Atmospheric reactions of sulfur dioxide are exceptionally complex^45^, causing sulfur species in the atmosphere to be oxidized into sulfuric acid. The initial step in the generation of sulfuric acid is the uptake of atmospheric SO_2_ into cloud water droplets, which is followed by its oxidation. It is expelled from the atmosphere during rain events. SO_2_ and SOx compounds contribute to acid rain, which can harm the ecosystems and result in corrosion of infrastructure. The SOx reacts with other compounds in the atmosphere to form little particles, which are the source of pollutions such as haze. The main source of sulfur dioxide emissions into the atmosphere originates from the consumption of petroleum products by control plants, transport load, combustion of fuel with high sulfur content, just like those originated from volcanoes such as reduced S species (COS, H_2_S and CS_2_)^46^.

SO_2_ and HO_2_ with EHP are significant atmospheric species. The reaction of sulfur dioxide with hydroperoxyl anion undergoes the formation of a SO_2_–HO_2_ complex assembled through van der Waals forces, and this process is a barrierless association. In pathway **E2** the C–O bond in EHP is broken and the HO_2_ atoms are bonded to the S atom of SO_2_, forming the bisulfate (HSO_4_^−^) and ethyl cation. Figure 15 shows the reaction mechanism of pathways **D1**, **E1** and **E2**.

Table 4 shows the activation energies and Gibbs energies of activation for Pathways **D1**, **E1** and **E2** at the B3LYP/6–311++G(3df,3pd) level of theory. The highest activation energy of TSD1 is 234 kJ mol^−1^ at the M06-2X/6-31G(d) level of theory relative to the other methods. Moreover, the overall activation energies at the B3LYP/6-311++G(3df,3pd), M11/6-31G(d), and B3LYP/6-31G (2df,p) levels of theory are 195, 229 and 193 kJ mol^−1^, respectively. The activation energy of TSD1 at the APFD/6-31G(d) level of theory is 185 kJ mol^−1^, which perfectly matches the corresponding value obtained at the B3LYP/6-31G(d) level of theory. The addition of a diffuse function to the B3LYP/6-31G(d) level of theory modestly increases the activation energy by 11 kJ mol^−1^ for TSD1, while the addition of the polarization function leads to the same activation energy. Furthermore, increasing the Gaussian function of the B3LYP method leads to slightly higher energy of 234 kJ mol^−1^ at the M06-2X/6-31G(d) level of theory. However, the water phase reduces the energy barrier of the TSD1 to 184 and 166 kJ mol^−1^ with PCM and SMD solvation models, respectively. By comparison, the SMD model is better than PCM, which reduces the energy barrier by 29 kJ mol^−1^_._ According to the activation energy values given in Table 4, the APFD/6-31G(d) and B3LYP/6-31G(d) levels of theory are more convenient in comparison with the other methods for this reaction.

**Table 4:** Activation energies and Gibbs energies of activation for the Pathways **D1, E1** and **E2**. at B3LYP/6-311++G(3df,3pd) level of theory.

Theory/basis set	TSD1		TSE1		TSE2	
	E_a_	ΔG^‡^	E_a_	ΔG^‡^	E_a_	ΔG^‡^
B3LYP/6-31G(d)	184	203	10	23	204	190
B3LYP/6-311++G(3df,3pd)	195	216	7	19	197	199
M11/6-31G(d)	229	240	26	33	244	246
M06-2X/6-31G(d)	234	246	27	33	245	248
APFD/6-31G(d)	185	198	6	17	208	211
B3LYP/6-31G(*2*df,p)	193	211	2	17	197	203
SMD	166	188	19	20	201	201
PCM	184	209	14	11	201	202

The energy barrier of TSE1 at the M11/6-31G(d) and M06-2X/6-31G(d) levels of theory are nearly the same with an energy value of 26 kJ mol^−1^. The effect of solvent using PCM and SMD models for pathway E1 increases the overall activation energy to 19 and 14 kJ mol^−1^, respectively (Table 4). This is consistent with the quantum chemical studies on the thermochemistry of alkyl and peroxyl radicals^47^. Brinck et al. reported the enthalpies for C–H bonds in substituted methane’s, C–O bonds in peroxyl radicals, and O–H bonds in hydroperoxides based on DFT calculations at the B3LYP/6–311 + G(2df,2p) and B3LYP/6-31G (d,p) levels of theory. Their energy barrier values were calculated in the range of − 4 to 10 kJ mol^−1^. These values are in good agreement with our results calculated herein at the B3LYP/6-31G(d), B3LYP/6-31G(2df,p), and APFD/6-31G(d) levels of theory. In TSE2 the overall activation energy at the M06-2X/6-31G(d) is 245 kJ mol^−1^. The B3LYP results utilizing the M06-2X/6-31G(d) optimized geometries are in good agreement with the values calculated at M11/6-31G(d) (244 kJ mol^−1^). However, lower overall activation energies are obtained with values of 197 kJ mol^−1^ at both the B3LYP/6-311G++(3df,3pd) and B3LYP/6-31G (2df,p) levels of theory. B3LYP/6-311++G(3df,3pd) and B3LYP/6-31G(2df,p) levels of theory are the most sufficient in comparison with the other methods for this reaction. Moreover, the activation energy value of TSE2 is higher by 73 kJ mol^−1^ than the energy reported for the reaction of SO_2_ + HO_2_, which is 124 kJ mol^−1^ at the B3LYP/6-31G(d) level of theory^48^.

### The oxidation reaction of EHP with ammonia (pathways F1 and F2)

Ammonia is the most inexhaustible environmental gas, which is a significant part of complete receptive nitrogen. It assumes a significant role in the arrangement of barometrical particulate issue and the statement of nitrogen in the atmosphere. Accordingly, the expansion in NH_3_ outflows contrarily impacts natural wellbeing just as environmental change. In this study, the oxidation response of NH_3_ with EHP has been explored to decide the most plausible mechanism. Pathway **F1** is a two-step reaction mechanism. The initial step includes an oxygen–oxygen bond cleavage in EHP, with oxygen moving from an ethyl hydroperoxide. The distal oxygen is attacked by the nucleophilic substrate (:NH_3_) with a direct SN2 type relocation of the peroxy oxygen. The second step involves 1,2-proton shift from amine to the adjacent alkoxide group, in which hydroxyl amine and ethanol are created through TSF1.

**Figure 16:**
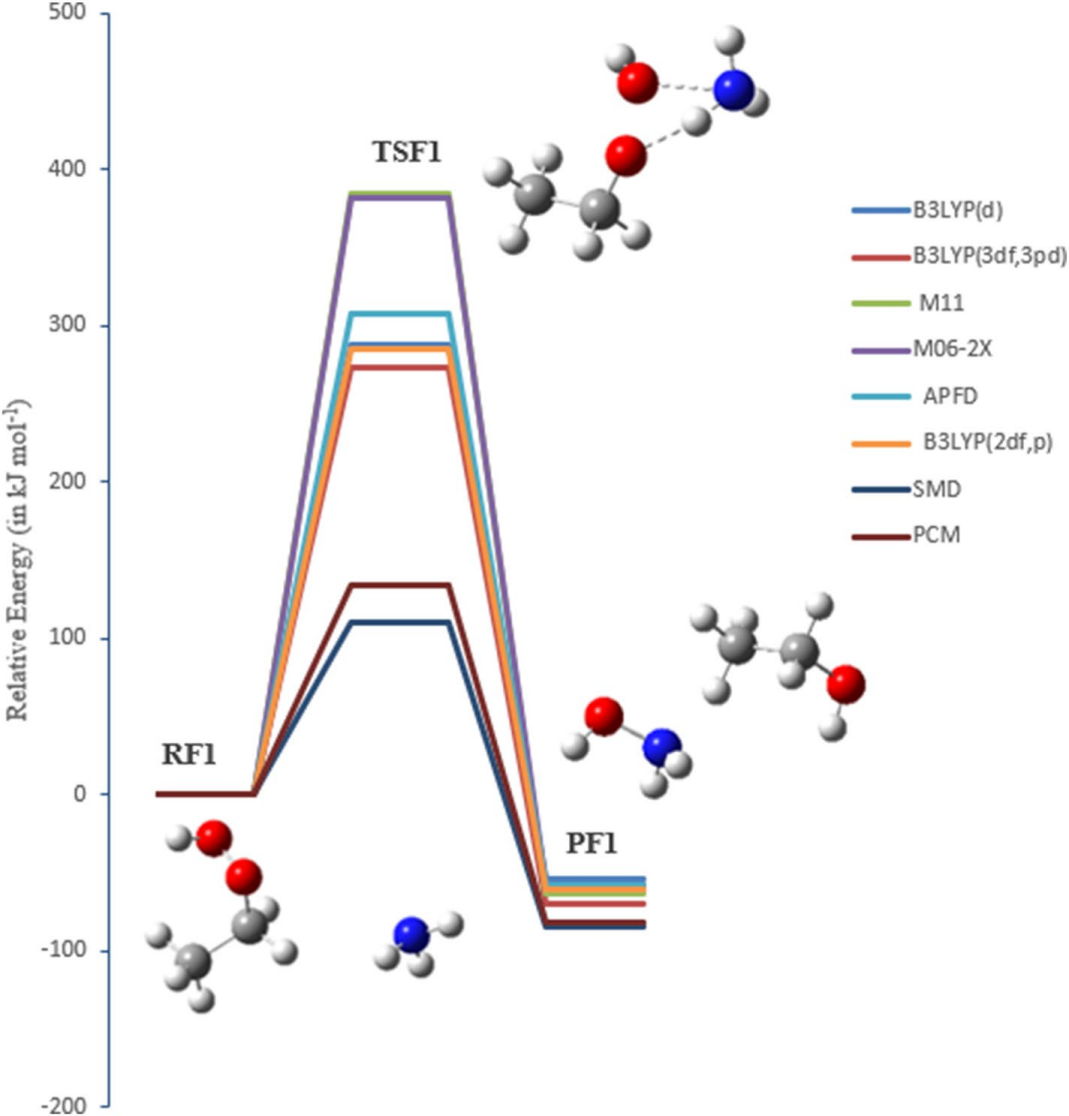
PED for the reaction of EHP with NH_3_ (pathway **F1**). Relative energies at several methods are given in kJ mol^−1^.

**Figure 17:**
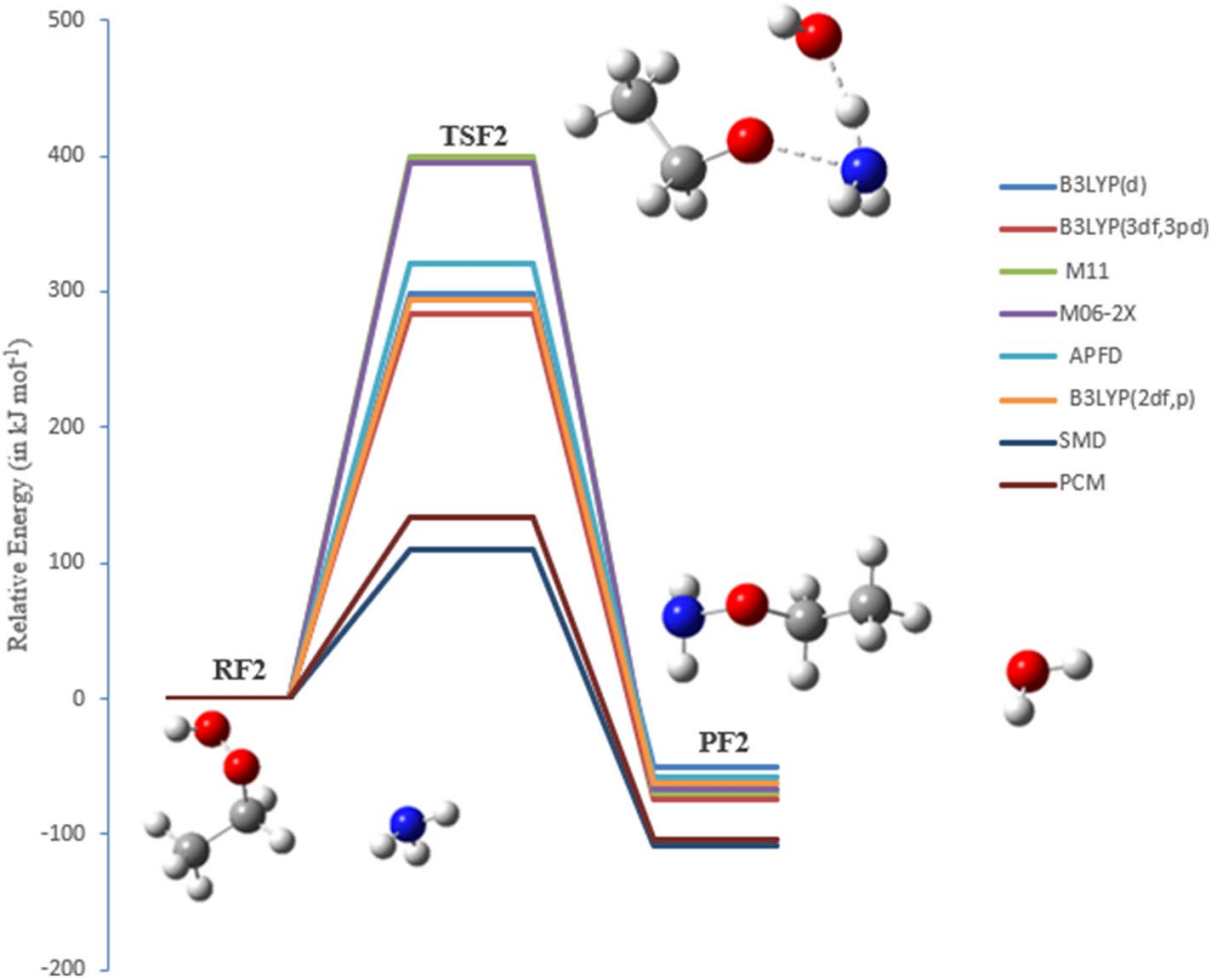
PED for the reaction of EHP with NH_3_ (pathway **F2**). Relative energies at several methods are given in kJ mol^−1^.

In pathway **F2**, the O–OH bond is cleaved. At that point, hydrogen migration from NH_3_ to the terminal oxygen occurs, resulting in H_2_O elimination. Subsequent to this, the nitrogen is connected with the distal oxygen, resulting in the formation of *O*-ethyl hydroxylamine and water (Fig. 16). There is a significant change in the O–OH bond, where the bond length is increased by 0.90 and 0.81 Å for pathways **F1** and **F2**, respectively. The bond length of TSF1 in O–OH is increased from 1.450 to 2.360 Å, and in TSF2 from 1.450 to 2.251. As can be seen, there is an insignificant difference of 0.04 Å in the N–O bond formation, where the bond length is 2.205 Å in TSF1, and 2.200 Å in TSF2. The length of the hydrogen bond ranges from 0.97 to 1.0 Å, which is increased by 0.03 and 0.07 Å, for pathways **F1** and **F2**, respectively **(**Figs. 16, 17). The bond lengths are reliable with the oxidation of amines with alkyl hydrogen peroxide^47^.

The activation energy of (TSF1) is 273 kJ mol^−1^, while the overall activation energy for (TSF2) is 284 kJ mol^−1^ at the B3LYP/6-311++G(3df,3pd) level of theory, Table 5. Along this line, the potential barrier of the separation should be high, and the reaction may not be favored in terms of energy. This is because of the hydrogen bond that stabilizes the pre-reactive complex compared with the reactants. Table 5 shows that lower energy values are obtained for the two pathways utilizing the B3LYP method with various basis sets; 6-31G(d), 6-31G (2df,p), and 6-311++G(3df,3pd). It is worthwhile mentioning that the addition of a diffuse function to the B3LYP method decreases the activation energy, while the addition of a polarization function increases the barriers. The overall activation energies of TSF1 at the B3LYP/6-31G(d) and B3LYP/6-31G (2df,p) levels of theory are 287, and 285 mol^−1^, respectively. In addition, the lower overall energy value of 273 kJ mol^−1^ is obtained at the B3LYP/6-311++G(3df,3pd) level of theory. The activation energy at the M06-2X/6-31G(d) is 382 kJ mol^−1^. The DFT results utilizing the M11/6-31G(d) basis sets are in good agreement with the M06-2X/6-31G(d) value of 384 kJ mol^−1^. By comparison of the activation energy of the 1,2-proton transfer (TSF1) and H_2_O elimination (TSF2), pathway **F1** is more plausible than pathway **F2**. The two pathways differ in activation energy by less than 11 kJ mol^−1^, as calculated at different levels of theory. There is a significant solvent effect (PCM and SMD models) on pathways **F1** and **F2 (**Figs. 16, 17). The overall activation energy is reduced to 111 and 113 kJ mol^−1^, respectively, as a result of solvation.

**Table 5:** Activation energies and Gibbs energies of activation for the reaction of EHP with NH_3_, Pathways **F1** and **F2**, at B3LYP/6–311++G(3df,3pd).

Theory/basis set	TSF1		TSF2	
	E_a_	ΔG^‡^	E_a_	ΔG^‡^
B3LYP/6-31G(d)	287	298	298	303
B3LYP/6–311++G(3df,3pd)	273	287	284	292
M11/6-31G(d)	384	393	399	405
M06-2X/6-31G(d)	382	388	395	399
APFD/6-31G(d)	307	315	320	324
B3LYP/6-31G(*2*df,p)	285	296	294	299
SMD	111	124	111	124
PCM	133	148	133	148

### The bimolecular reaction of chloromethane (CH_3_Cl) with EHP (pathways G1, G2 and H1)

Chloromethane (CH_3_Cl) considered as the most abundant naturally created chlorine-containing organic compound in the atmosphere. It significantly affects chlorine chemistry in the environment and is involved in various catalytic cycles responsible for the deterioration of the stratospheric ozone layer. Regardless of its significance, the understanding of the atmospheric budget of CH_3_Cl is still lacking. Bimolecular nuclear substitution (SN2) reactions are of the basic organic reactions and have been widely explored from both experimental and theoretical studies, particularly, halogen exchange reactions^49,50^.

Even though the dynamics of halogen exchange interactions were theoretically known, the mechanism of SN2 reactions in molecular ions (OH^−^ and HOO^−^) has not been fully comprehended. This is because the dimension, which must be included in the calculations, will increase significantly as the number of atoms in the reaction system increases^51^. There are three primary possible pathways for the reaction of chloromethane with ethyl hydroperoxide, denoted as pathways **G1**, **G2**, and **H1**.

**Figure 18:**
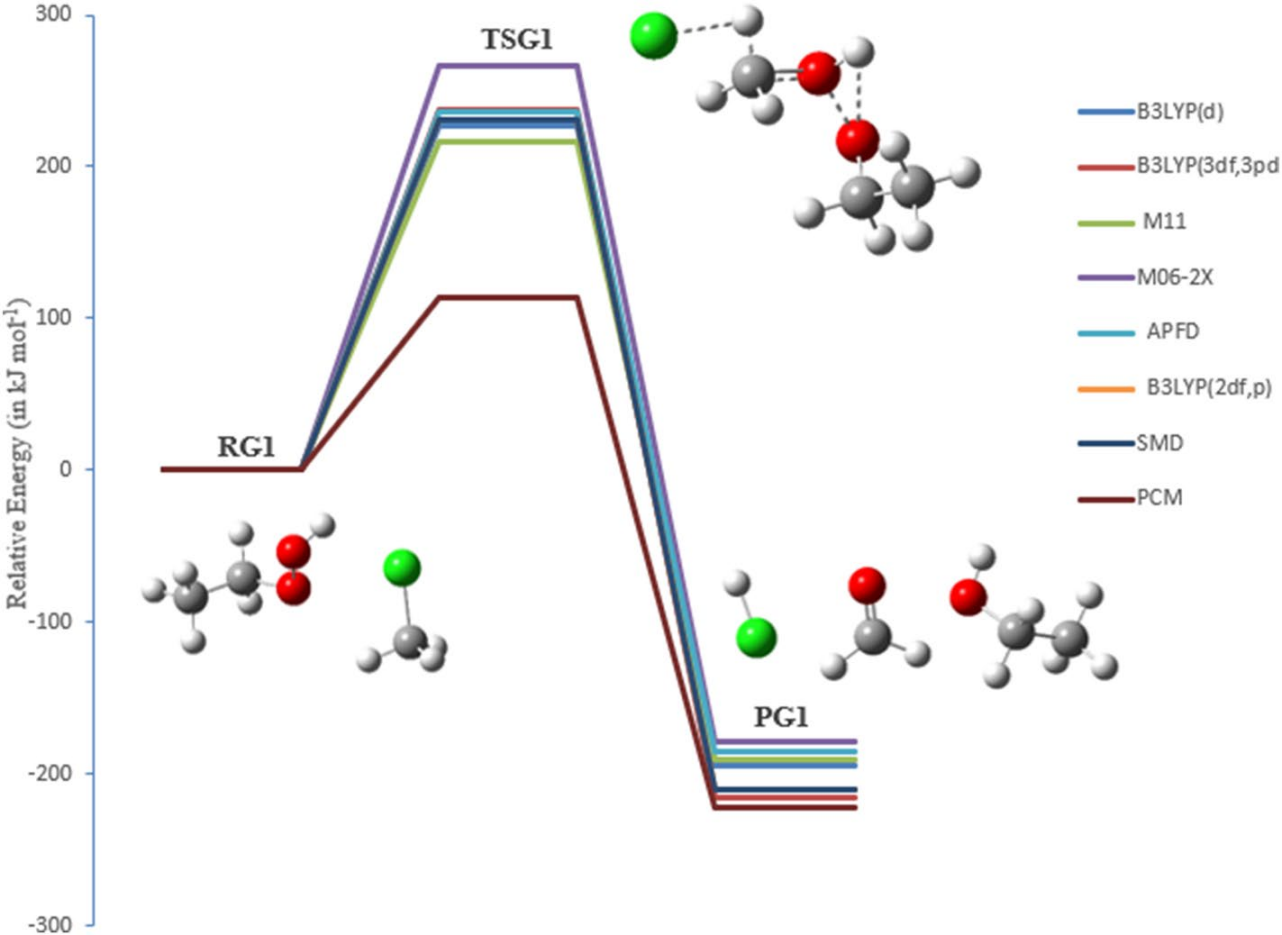
The PED for the reaction of EHP with CH_3_Cl (Pathway **G1**). Energies calculated at several levels of theory.

In the reaction mechanism of pathway **G1**, OH radicals are regenerated through the gas-phase reaction of CH_3_CH_2_OOH with chloromethane via transition state TSG1. There are impressive changes on the bond lengths of TSG1. As shown in Fig. 18, there are some noteworthy changes in the bond lengths and torsion angles. Particularly, the decomposition of EHP by photolysis happens in a concerted bond-breaking step of the O–OH group. OH radical is essentially created, where the bond is elongated from the molecule by 0.220 Å to become 1.630 Å, forming ethylperoxy CH_3_CH_2_OO and hydroxyl radical OH. At that point, methyl and Cl radicals are formed through the photolysis of CH_3_Cl, with the C–Cl bond increased from 1.830 Å to 2.641 Å. Additionally, the ethyl peroxy radical yields formaldehyde and CH_3_ radical, with a C=O double bond formed at 1.210 Å. The Cl radical is further attached to the hydrogen radical, form HCl with a bond length of 0.961 Å. Finally, the CH_2_CH_3_ radical is associated with the OH radical, resulting in the formation of ethanol (PG1).

Reaction mechanism of Pathway G1:


It ought to be noticed that the O–O bond length is in the range of 1.453 Å, and C–Cl bond length is in the range of 1.858 Å at all levels of theory. This is predictable with the detailed geometries in the literature^52^.

**Figure 19:**
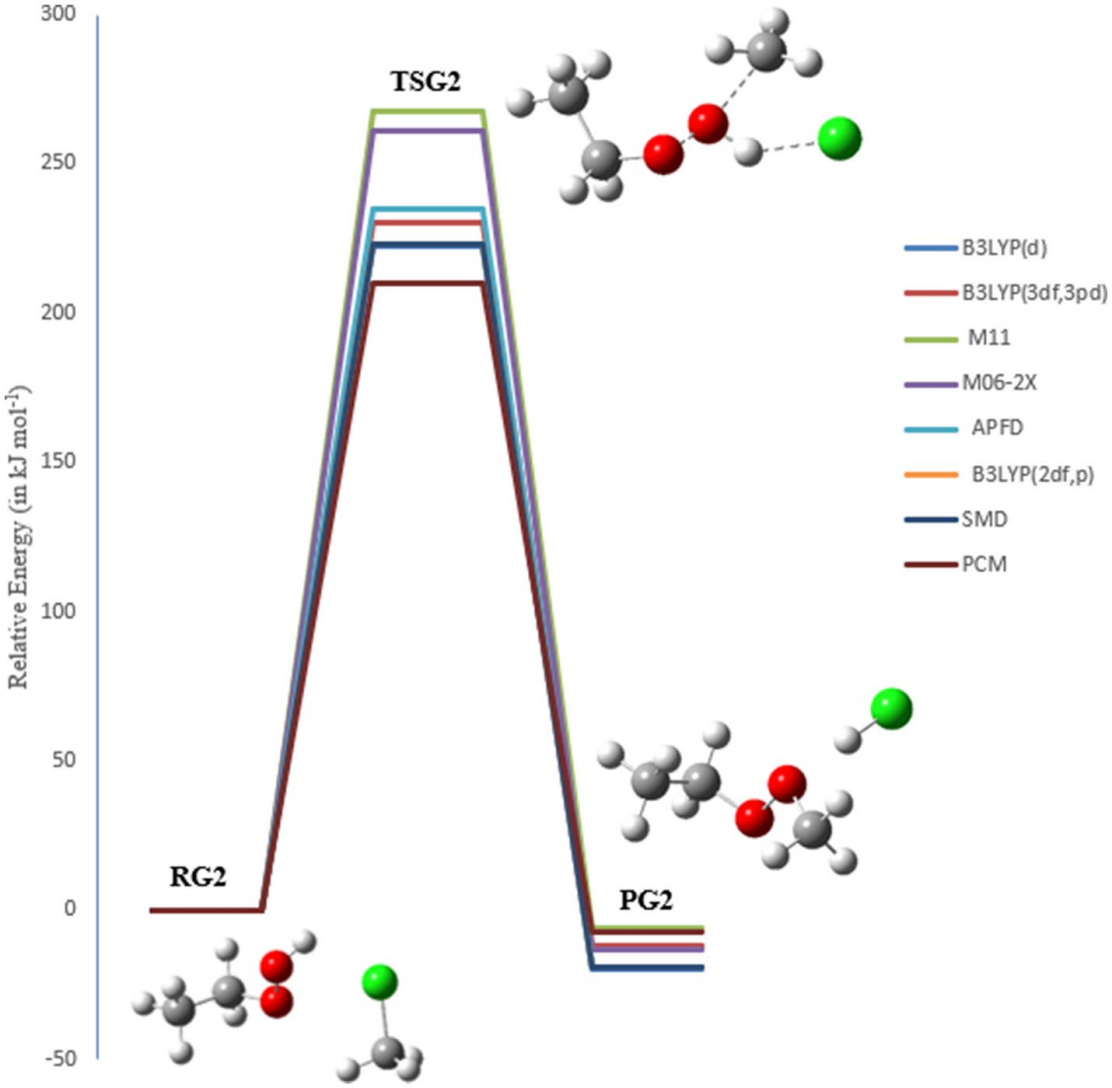
The PED for the reaction of EHP with CH_3_Cl (Pathway **G2**). Energies calculated at several methods.

**Figure 20:**
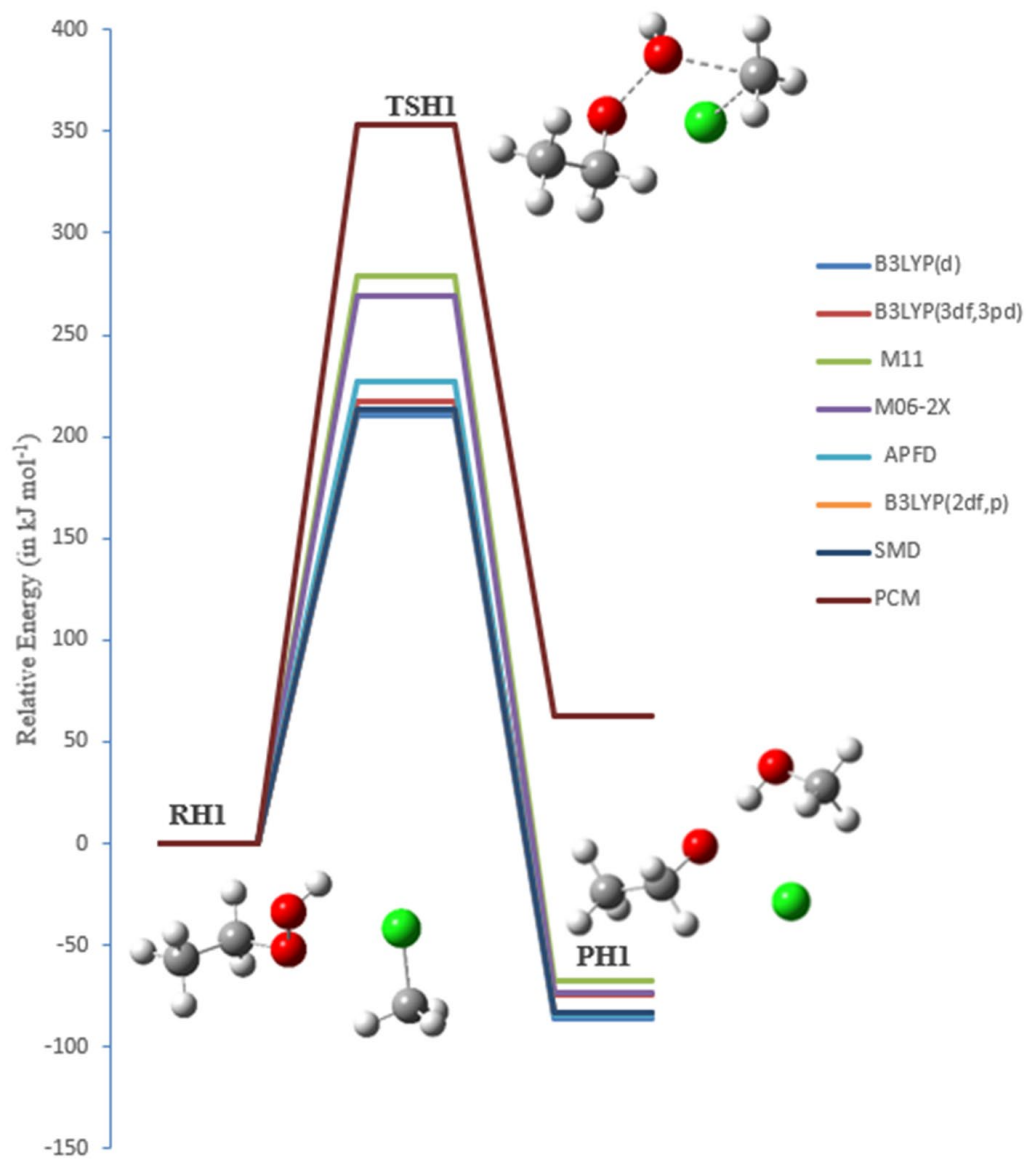
The PED for the reaction of EHP with CH_3_Cl (Pathway **H1**). Energies calculated at several methods.

A hydrogen abstraction reaction started by chlorine atom and methyl substitutions was investigated (pathway **G2**). The SN2 reaction of molecular ion OH^−^ with CH_3_Cl was studied (pathway **H1**). This leads to the formation of the ethyl-methyl-peroxide through TSG2 and methanol through TSH1 as shown in Figs. 19 and 20. There are some significant changes in bond lengths and torsion angles. TSG2 shows a C–Cl bond in chloromethane elongated from 1.80 to 2.60 Å to form Cl and methyl radical, which are in great concurrence with the work reported by Evanseck et al., This study investigates that the geometry of the transition state shows an extension of the C–Cl bond length to 2.10 from 1.79 Å in methyl chloride^47^, which is accompanied with a slight increase in the bond length of O–H bond length by 0.110 Å to 1.00 Å. H and CH_3_CH_2_OO radicals are then formed. Likewise, the distance between H–Cl is decreased by 0.50 Å, and the distance between the methyl radical and the terminal oxygen in CH_3_CH_2_OO radical is reduced from 3.63 Å to 2.10 Å. The structure of TSH1 reveals that there is an increase in the C–Cl bond length from 1.80 to 2.41 Å, and there is an increase in the O–OH bond length by 0.721 Å to 2.11 Å. Furthermore, the C–O bond is decreased by 1.810 Å, resulting in methanol. The SN2 reaction of OH^−^ with CH_3_Cl is shown below:

$$ \begin{array}{*{20}l} {{\text{OH}}^{ - } \, + \,{\text{CH}}_{{3}} {\text{Cl}}\, \to \,{\text{CH}}_{{3}} {\text{OH}}\, + \,{\text{Cl}}^{ - } } \hfill \\ {{\text{HOOCH}}_{{2}} {\text{CH}}_{{3}} \, + \,{\text{CH}}_{{3}} {\text{Cl}}\, \to \,{\text{CH}}_{{3}} {\text{OH}}\, + \,{\text{Cl}}^{ - } \, + \,{\text{CH}}_{{3}} {\text{CH}}_{{2}} {\text{O}}^{ + } } \hfill \\ \end{array} $$

Table 6 shows the energy barriers obtained for pathways **G1**, **G2**, and **H1** using the B3LYP/6-31G (2df,p), which are 231, 223, and 214 kJ mol^−1^, respectively. Ultimately, the radicals of CH_3_Cl and CH_3_CH_2_OOH are formed via TSG1, TSG2 and the substitution reaction via TSH1. The overall activation energies of TSG1, TSG2 and TSH1 are 216, 268 and 279 kJ mol^−1^, respectively, at the M11/6-31G(d) level of theory.

**Table 6:** Activation energies, enthalpies of activation, and Gibbs energies of activation for the reaction of EHP with CH_3_Cl (in kJ mol^−1^) at 298.15 K (Pathway **G1, G2 and H1**).

Theory/basis set	TSG1		TSG2		TSH1	
	E_a_	ΔG^‡^	E_a_	ΔG^‡^	E_a_	ΔG^‡^
B3LYP/6-31G(d)	227	243	222	235	210	229
B3LYP/6-311++G(3df,3pd)	237	250	230	241	217	239
M11/6-31G(d)	216	232	268	267	279	300
M06-2X/6-31G(d)	266	269	261	270	269	286
APFD/6-31G(d)	236	250	235	246	227	243
B3LYP/6-31G(*2*df,p)	231	246	223	235	214	232
SMD	114	126	210	219	353	357
PCM	118	128	209	222	355	364

In TSG1, the overall activation energy of 266 kJ mol^−1^ at M06-2X/6-31G(d) level of theory is the highest, relative to the other methods. The overall activation energies at the B3LYP/6-31G(d), B3LYP/631G (2df,p), and B3LYP/6-311++G(3df,3pd) levels of theory are 227, 231, and 237 kJ mol^−1^, respectively.

In TSG2, the activation energy at the M11/6-31G(d) level of theory with a value of 268 kJ mol^−1^ is higher than the energies calculated by other DFT methods. The differences in the activation energies for pathways **G1**, **G2**, and **H1** using DFT functionals (see Table 6) amount to 39, 46, and 59 kJ mol^−1^, respectively. The energy values at the B3LYP/6-31G(d), B3LYP/6-31G (2df,p), and B3LYP/6-311++G(3df,3pd) levels of theory are 222, 223 and 230 kJ mol^−1^, respectively. As for TSH1, the activation energy of TSH1 at the B3LYP/6-311++G(3df,3pd) level of theory is 217 kJ mol^−1^, which is similar to the results of the B3LYP/6-31G(d) and B3LYP/6-31G (2df,p) levels of theory. The overall activation energy of 279 kJ mol^−1^ at M11/6-31G(d) level of theory is the highest, relative to the other methods. The overall activation energies at the M06-2X/6-31G(d), APFD/6-31G(d), levels of theory are 269 and 227, kJ mol^−1^, respectively.

Interestingly, the least-expensive method B3LYP/6-31G(d) yielded the lowest barriers for pathways **G1**, **G2**, and **H1**, with values 227, 222, and 210 kJ mol^−1^, respectively.

It is worth mentioning that the use of diffuse and Gaussian functions in the calculations of TSG1 and TSG2 with the B3LYP method leads to an increase in the energy barrier. The addition of a diffuse function in the B3LYP method increases the energy barrier, while the addition of a polarization function (more Gaussian functions) increases it. By comparison, the relative energies of pathways **G2** and **H1** are − 20 and − 86 kJ mol^−1^ at the B3LYP/6-31G(d) level of theory, respectively. The barrier for methyl transfer reaction was found to be − 21.1 kJ mol^−1^ at B3LYP/6–31 + G, which is in excellent agreement with B3LYP/6-31G(d) value of − 20 kJ mol^−1^^53^. Moreover, the relative energy barrier reported by Evanseck et al.^52^ for the reaction of OH^−^ with CH_3_Cl is − 81.4 kJ mol^−1^ at the B3LYP/6-31G(d) level of theory, which is in good agreement with the B3LYP/6-31G(d) value of − 86 kJ mol^−1^.

The water phase significantly drops the energy barriers of both of TSG1 and TSG2 to 114 and 210 kJ mol^−1^ at SMD, and to 118 and 209 kJ mol^−1^ at PCM, respectively. It is worth noting here that the solvent exerts a significant impact on the energetics of pathway H1. The overall activation energy in the aqueous phase is increased to 163 kJ mol^−1^, while a few locales are accessible for strong solute–solvent hydrogen-bonding interactions.

### The thermodynamic parameters of the reaction of methane with Criegee intermediate

The thermodynamic parameters (∆H and ∆G) for the reaction of methane with Criegee intermediate along with its proposed reactions are studied at all levels of theory. The reactions of methane with Criegee intermediate in unimolecular and bimolecular manners were found to be exceptionally exothermic and exergonic at all levels of theory. This is in line with the reported atmospheric reactions of the Criegee intermediate with methane^7,43^.

The bimolecular reaction of the EHP with SO_2_, pathway **E2**, is endothermic and endergonic at all levels of theory. In this manner, the creation of bisulfate HSO4^−^ and ethyl cation is not favored. In view of these results, we can conclude that the pathways **A1**, **B1**, and **B2** incur the lowest thermodynamic parameters values and they are therefore more spontaneous and plausible reactions to occur in the atmosphere.

The solvation effect is supported thermodynamically by that of the overall Gibbs energy of the reaction in water is lower than the reaction in the gas phase. For pathways **A1**, **A2**, **B1**, **B2**, **C3**, **G2**, and **H1**, the solvation effect leads to increased ∆G values compared with those of the gas phase, indicating that solvation does not essentially promote the thermodynamic driving forces in these reactions. Pathways **C1**, **C2**, **D1**, **E1**, **E2**, **F1**, **F2**, and **G1**, on the other hand, are more thermodynamically favorable in water, as their ∆G values are decreased by the solvation effect.

## Conclusions

In this study, a comprehensive computational investigation for the gas-phase reaction of methane with Criegee intermediate has been carried out utilizing accurate quantum chemical DFT calculations. Four significant pathways for the unimolecular reaction of ethyl hydroperoxide (EHP), and eleven for the bimolecular reactions with H_2_, H_2_O, CO_2_, CH_4_, SO_2_, NH_3_, and CH_3_Cl were studied. The potential energy diagram (PED) for each of the reaction pathway was clearly mapped out utilizing the B3LYP, M06-2X, M11 and APFD methods. The thermodynamic (ΔH and ΔG) and kinetic parameters (E_a_, ΔH^ǂ^, and ΔG^ǂ^) were calculated, using the DFT methods, for each proposed pathway. The connections of the TS's with the I's, R's, and P's of every pathway have been confirmed using the intrinsic reaction coordinate (IRC) calculations. The results of the atmospheric reactions of methane with Criegee intermediate are sensitive to the basis sets. Moreover, the reactions are all exothermic, except in the case of the bimolecular reaction of the EHP with SO_2_ leading to the formation of bisulfate (Pathway **E2**), where the reaction is endothermic. The reaction of methane with Criegee leads to the formation of EHP. It should be mentioned that the activation energies for eight pathways have calculated using a higher level of theory at G4MP2. It was found that the barriers calculated at the B3LYP/6-311++G(3df,3pd) level of theory are very comparable to the most computationally expensive methods such as Gaussian-n theories (G4MP2) differing by no more than 3–17 kJ mol^−1^, see Table S2 in the SI. Therefore, B3LYP/6-311++G(3df,3pd) level of theory will be reliable and a good choice to study such systems compared to the most computationally expensive method, G4MP2.

Along these lines, the EHP breaks down to create various products. The formation of acetaldehyde, methanol, and hydrogen peroxide were found more likely to occur. From that point, the EHP undergoes a series of complex unimolecular and bimolecular reactions. Conformational changes were found during the first and second TS's in some cases. The photochemical reactions of methane with ethyl hydroperoxide are energetically more favored compared to all other pathways. Pathway **E1** has the lowest overall activation energy in the gas phase of 7 kJ mol^−1^ at B3LYP/6-311++G(3df,3pd) theory. The use of the implicit solvation models (PCM and SMD) did not decrease the barrier height of pathway **E1**. This pathway shows a two-step mechanism. The initiation of a radical chain mechanism for the reaction of methane with EHP occurs under UV light irradiation. UV light causes the weak EHP bond to undergo homolytic cleavage to generate ethoxy and hydroxyl radicals, which start the chain process. In the propagation step the hydroxyl radical abstracts methane hydrogen to form H_2_O and a methyl and ethoxy radical. For most of the proposed mechanisms, it has been found that the PCM solvation model gives a higher activation barrier than that of the SMD solvation model. The results calculated at the B3LYP/6-311++G(3df,3pd) level of theory are in excellent agreement with APFD method for all pathways investigated.

## Supplementary information

Supplementary Information
