# Supplementary material for: Computational mechanistic study of the unimolecular dissociation of ethyl hydroperoxide and its bimolecular reactions with atmospheric species
Source: Sci Rep. 2020 Sep 14;10:15025. doi: 10.1038/s41598-020-71881-3 (PMC7490386; doi:10.1038/s41598-020-71881-3)

Supplementary Information

Computational Mechanistic Study of the Unimolecular Dissociation of Ethyl Hydroperoxide and its Bimolecular Reactions with Atmospheric Species

Mansour H. Almatarneh[1,2,*], Asmaa Alnajajrah[1], Mohammednoor Altarawneh[3], Yuming Zhao[2], Mohammad A. Halim[4]

[1] *Department of Chemistry, University of Jordan, Amman 11942, Jordan.*

[2] *Department of Chemistry, Memorial University, St. John's, NL A1B 3X7, Canada.*

[3] Department of Chemical and Petroleum Engineering, United Arab Emirates University, Al-Ain, *15551, UAE*

[4] *Department of Physical Sciences, University of Arkansas, Fort Smith, Arkansas, 72913, USA*

*** Correspondence:**

M. H. Almatarneh: m.almatarneh@ju.edu.jo

Contents:

Figure S1. Criegee intermediate structure representation, 'R' is an arbitrary functional group.

Figure S2. Optimized structures for pathways **D1, E1**, and **E2**.

Table S1. Selected bond lengths (Å), bond angles (°), and dihedral angles (Torsions) (°) of the optimized geometry of reactant at B3LYP/6-311G++(3df,3pd) level of theory.

Table S2. Comparison of the selected activation energies of proposed pathways that have calculated at B3LYP/6-311G++(3df,3pd) and G4MP2 methods.

Table S3. Thermodynamic Parameters for the reaction of methane with Criegee intermediate (in kJ mol^{-1}) at 298.15 K, Pathways **A1** → **H1.**

- **Cartesian coordinates for all investigated pathways.**

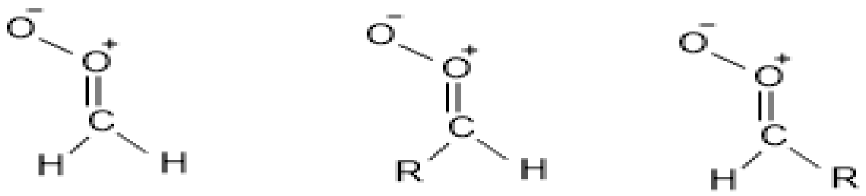

Simplest CI | *Syn*-CI | *Anti*-CI

Figure S1. Criegee intermediate structure representation, 'R' is an arbitrary functional group.

Table S1. Selected bond lengths (Å), bond angles (°), and dihedral angles (Torsions) (°) of the optimized geometry of reactant at B3LYP/6-311G++(3df,3pd) level of theory.

	Bond lengths (Å)
C=O<br>C-H, (C1-H2, H3)<br>(C6- H7, H8, H9, H10)<br>O-O, (O4-O5)	1.520<br>1.083<br>1.089<br>1.348
	**Bond angles (°)**
C-H-H, in methane (C6-H9-H10)<br>(C6-H7-H8)<br>C-H-H, in CI (C1-H2-H3)<br>C-O-H, (C1-O5-H2)<br>(C1-O5-H3)<br>C-O-O, (C1-O4-O5)	109.453<br>109.154<br>125.234<br>119.375<br>115.391<br>119.185
	**Dihedral angles**
O4–O5-C1–H2	0.312

Table S2. Comparison of the selected activation energies of proposed pathways that have calculated at B3LYP/6-311G++(3df,3pd) and G4MP2 methods.

**Theory/Basis Set**	**TS1A**	**TS2A1**	**TS2A2**	**TS2B1**	**TS2B2**	**TSC1**	**TSD1**	**TSF2**
**Ea (kJ mol^{-1})**	E_a	E_a	E_a	E_a	E_a	E_a	E_a	E_a
**B3LYP/6-311++G(3df,3pd)**	106	196	235	234	234	313	195	284
**G4MP2**	103	179	251	239	244	329	195	294

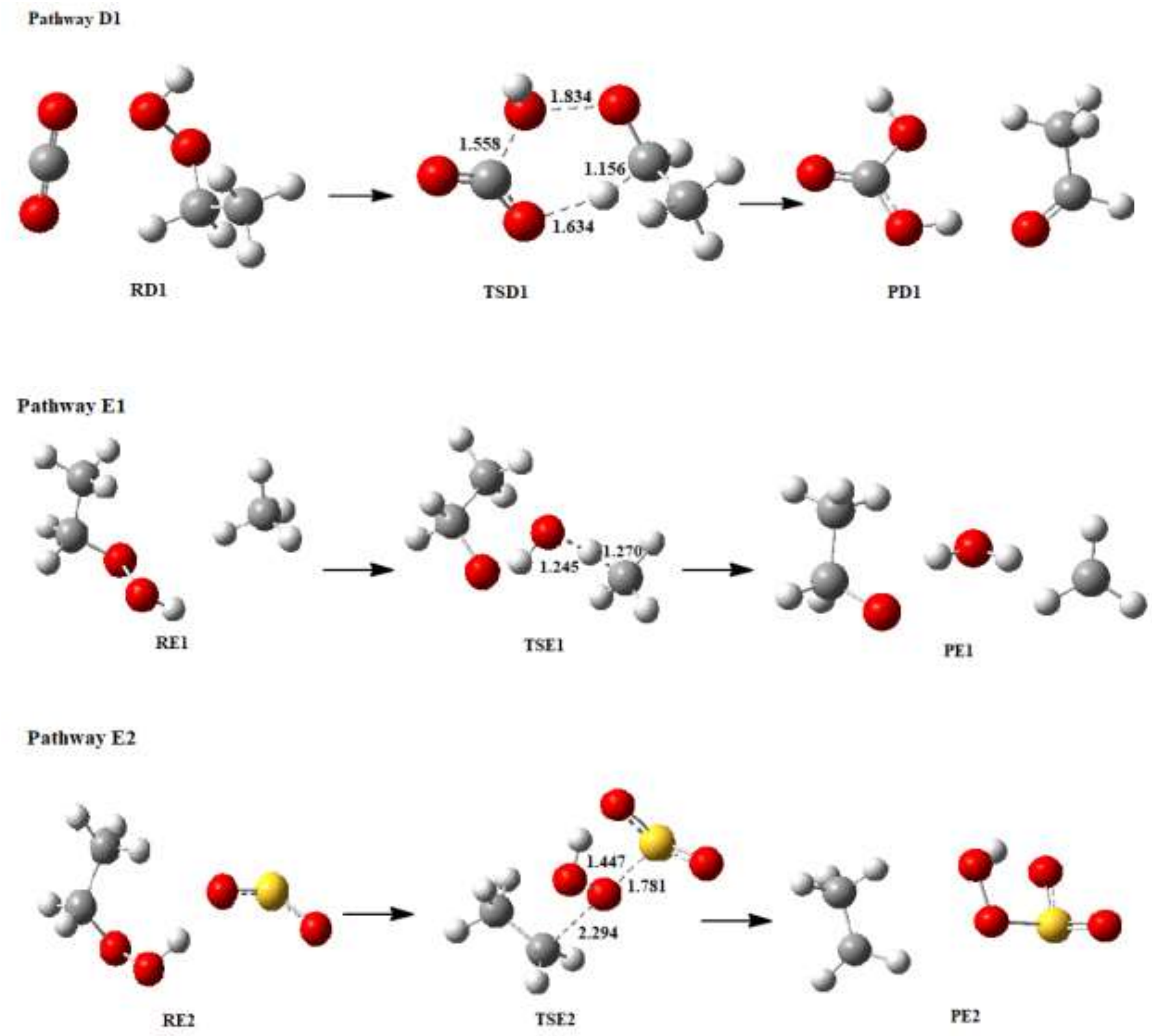

Figure S2. Optimized structures for pathways **D1, E1**, and **E2**.

Table S3: Thermodynamic Parameters for the reaction of methane with Criegee intermediate (in kJ mol^{-1}) at 298.15 K, Pathways **A1** → **H1.**

Reaction/Parameters	B3LYP/6-31G(d)	B3LYP/6-311G++(3df,3pd)	M11/6-31G(d)	M06-2X/6-31G(d)	APFD/6-31G(d)	B3LYP/6-31G (2df,p)	SMD	PCM
**Pathway A1**								
ΔH	-396	-442	-430	-429	-410	-416	-440	-438
ΔG	-387	-435	-428	-424	-404	-407	-431	-417
**Pathway A2**								
ΔH	-400	-443	-443	-432	-414	-421	-439	-439
ΔG	-390	-435	-429	-428	-408	-410	-429	-414
**Pathway B1**								
ΔH	-328	-352	-357	-359	-334	-338	-327	-341
ΔG	-321	-339	-357	-354	-330	-331	-321	-315
**Pathway B2**								
ΔH	-74	-112	-98	-102	-82	-92	-98	-99
ΔG	-71	-102	-97	-98	-80	-89	-92	-75
**Pathway C1**								
ΔH	-273	-329	-306	-295	-288	-294	-350	-340
ΔG	-263	-321	-304	-293	-281	-298	-346	-335

**Pathway C2**								
ΔH	-42	-62	-50	-46	-46	-51	-74	-65
ΔG	-46	-65	-58	-51	-48	-55	-76	-69
**Pathway C3**								
ΔH	-62	-80	-70	-66	-68	-73	-97	-90
ΔG	-68	-83	-73	-69	-74	-77	-100	-94
**Pathway D1**								
ΔH	-222	-239	-242	-234	-228	-227	-278	-263
ΔG	-216	-231	-239	-229	-223	-221	-267	-256

**Reaction/Parameters**	B3LYP/6-31G(d)	B3LYP/6-311G++(3df,3pd)	M11/6-31G(d)	M06-2X/6-31G(d)	APFD/6-31G(d)	B3LYP/ 6-31G (2df, p)	SMD	PCM
**Pathway E1**								
ΔH	-25	-61	-30	-27	-32	-41	-69	-67
ΔG	-26	-58	-28	-26	-31	-40	-64	-66
**Pathway E2**								
ΔH	137	123	141	143	130	122	128	121
ΔG	101	108	138	142	127	116	104	105
**Pathway F1**								
ΔH	-55	-69	-63	-58	-58	-61	-84	-82
ΔG	-52	-65	-57	-55	-59	-59	-77	-74
**Pathway F2**								
ΔH	-50	-74	-70	-67	-58	-62	-108	-104
ΔG	-49	-74	-62	-62	-55	-60	-99	-96

Reaction/Parameters	B3LYP/6-31G(d)	B3LYP/6-311G++(3df,3pd)	M11/6-31G(d)	M06-2X/6-31G(d)	APFD/6-31G(d)	B3LYP/6-31G (2df, p)	SMD	PCM
**Pathway G1**								
ΔH	-195	-215	-190	-179	-186	-210	-222	-227
ΔG	-198	-225	-194	-192	-190	-214	-229	-233
**Pathway G2**								
ΔH	-20	-12	-6	-13	-19	-19	-7	-12
ΔG	-13	-5	-12	-12	-13	-13	2	1
**Pathway H1**								
ΔH	-86	-74	-67	-73	-84	-83	-62	-64
ΔG	-74	-64	-60	-63	-74	-72	-55	-46

Cartesian Coordinates:

R: Reactant ; TS: Transition State ; P: Product

Pathway A1

A1-R1-631gd

Charge = 0 Multiplicity = 1

C	0.18321	1.12056	0.26549
H	-0.11	0.92613	1.28917
H	0.0306	2.0694	-0.23829
O	0.87862	-0.98618	0.19806
O	0.8771	0.26561	-0.35775
C	-1.87676	-0.30067	-0.1047
H	-0.92007	-0.737	0.19426
H	-2.63295	-0.98225	0.30111
H	-2.12119	0.69652	0.26966
H	-1.94776	-0.29916	-1.19614

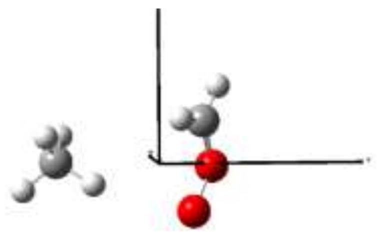

A1-TS1-631gd

Charge = 0 Multiplicity = 1

C	0.25953	0.46444	1.43597
H	0.16956	1.52817	1.36327
H	-0.45761	0.00358	0.78928
O	2.29488	-0.41441	2.0545
O	1.51984	0.10188	1.08556
C	-0.11702	-0.22046	3.63124
H	1.2991	-0.38037	3.02492
H	-0.1281	0.54318	4.38066
H	-1.18398	-0.21827	3.55062
H	-0.12949	-1.15037	4.16039

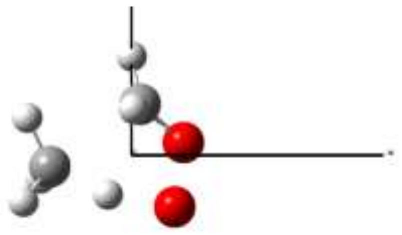

A1-P1-6-31gd

Charge = 0 Multiplicity = 1

C	-0.10139	0.95508	0.23545
H	0.00526	1.01832	1.32401
H	-0.22025	1.93008	-0.24428
O	0.79953	-1.02454	0.19763
O	0.94093	0.28935	-0.37095
C	-1.67674	-0.07553	-0.07197
H	-0.16953	-1.12339	0.14455
H	-1.88182	-0.95086	0.56108
H	-2.45372	0.65794	0.15243

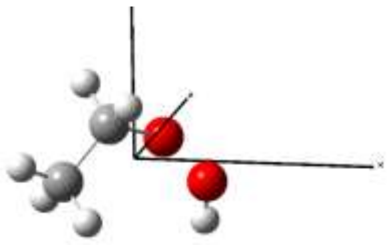

A1-R2-631gd

Charge = 0 Multiplicity = 1

C	-0.52588	0.20175	0.50369
H	0.06389	-0.63466	0.92571
H	-0.76542	0.80781	1.39425
O	1.58285	-0.51651	0.00586
O	0.36732	0.84868	-0.30635
C	-1.71705	-0.45636	-0.17972
H	2.49385	-0.51148	-0.3729
H	-1.37475	-1.03578	-1.0427
H	-2.20481	-1.13721	0.52734
H	-2.45938	0.27324	-0.52049

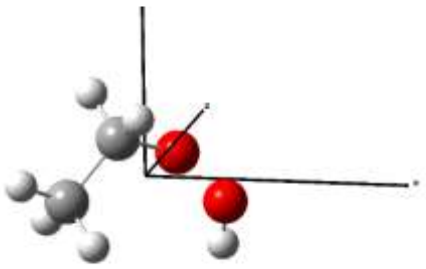

A1-TS2-631gd

Charge = 0 Multiplicity = 1

C	-0.47498	0.34461	0.38423
H	0.51434	-0.72425	0.91363
H	-0.75172	1.09347	1.09663
O	1.67798	-0.69431	0.24662
O	0.47929	0.74274	-0.53873
C	-1.79218	-0.19443	-0.204
H	2.00267	-1.43808	-0.2662
H	-1.57584	-0.95208	-0.92792
H	-2.38913	-0.61067	0.58041
H	-2.32678	0.60493	-0.67319
H	-1.72834	-0.33793	-1.1332

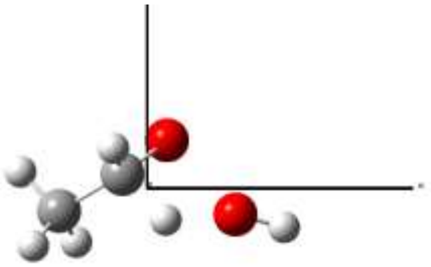

A1-P2-631gd

Charge = 0 Multiplicity = 1

C	-2.09674	0.37791	-0.01625
H	-2.36198	0.81886	0.95795
H	-2.69383	-0.51999	-0.18048
H	-2.3346	1.14639	-0.76592
C	-0.61989	0.09715	0.00192
H	0.03896	0.99151	0.03106
O	-0.12017	-1.01298	-0.003
O	-0.16725	-1.37558	2.56989
H	-0.80255	-2.09198	2.73591
H	0.10081	-1.53496	1.65283

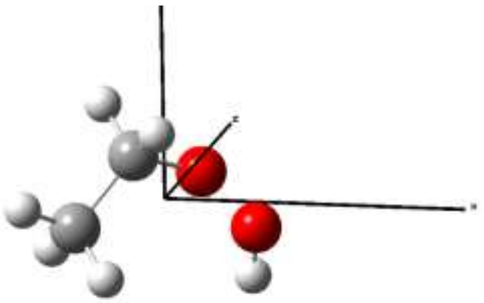

Pathway A2

A2-R-631gd

Charge = 0 Multiplicity = 1

C	1.01115	-0.34011	0.28122
H	1.13688	-0.45851	1.3696
H	1.95275	-0.65033	-0.23156
O	-1.61648	0.33636	0.09167
O	0.02613	-1.09146	-0.25165
C	0.82455	1.12623	-0.12545
H	-1.86046	-0.60316	0.17645
H	-0.22124	1.25625	0.18252
H	1.4937	1.81639	0.39518

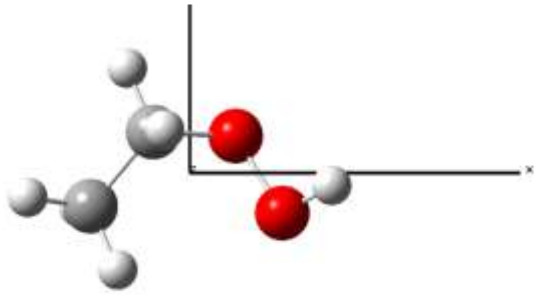

H	0.88106	1.26329	-1.20784

A2-Ts-631gd

Charge = 0 Multiplicity = 1

C	3.31998	-0.87292	0.21871
H	3.05883	-0.85001	1.2561
H	4.25341	-1.38164	0.09704
O	-0.28803	-0.14927	-0.17606
O	2.3029	-1.56134	-0.51377
C	3.4477	0.56768	-0.31042
H	-0.92596	-0.74302	0.22656
H	2.51427	1.0764	-0.18875
H	4.20873	1.08279	0.23766
H	3.70885	0.54477	-1.34781

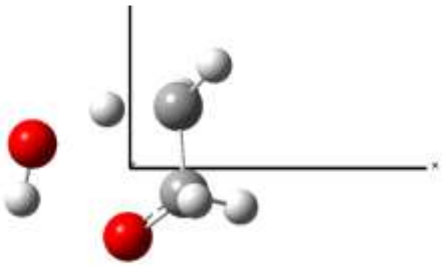

A2-p-6-31gd

Charge = 0 Multiplicity = 1

C	0.94732	-0.34429	0.28335
H	1.19255	-0.46429	1.36106
H	1.96115	-0.37273	-0.27553
O	-1.63263	0.42258	0.10584
O	0.09605	-1.1531	-0.25896
C	0.84696	1.09693	-0.13015
H	-1.49778	-0.53731	0.11448
H	-0.74629	0.85209	0.17362
H	1.39951	1.8559	0.42541
H	0.71333	1.29865	-1.19298

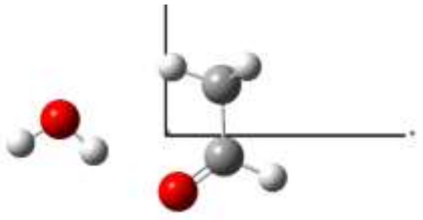

Pathway B1

B1-R-631gd

Charge = 0 Multiplicity = 1

C	0.3688	-0.99815	-0.08662
H	0.42244	-1.45313	-1.08337
H	0.38554	-1.71411	0.74385
O	-0.08696	1.25234	-0.06199
O	1.21587	0.03383	0.09613
C	-1.4992	-0.50615	0.04466
H	0.18692	2.19373	0.05565

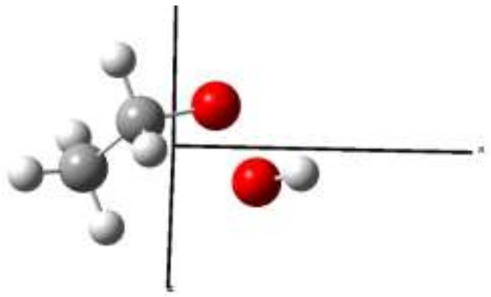

B1-TS-631gd

Charge = 0 Multiplicity = 1

C	-0.48471	1.24171	-0.2523

H	-0.81431	1.94699	0.48176
H	-0.89015	1.52527	-1.20104
O	1.00207	-0.69895	0.23316
O	0.82915	1.16925	-0.28755
C	-1.10557	-0.94857	0.32117
H	1.45678	-1.26601	-0.39396
H	-1.06316	-1.75105	1.02765
H	-2.03138	-0.65257	0.76856
H	-1.41949	-1.45673	-0.5666
H	-1.90827	0.26272	-0.60204
H	-1.86624	-1.49033	-0.25423
H	-1.67436	-0.30119	1.09817

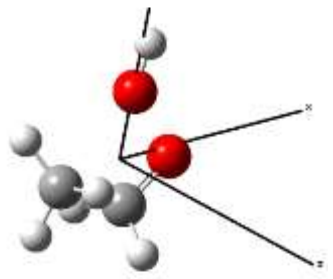

B1-P-631gd

Charge = 0 Multiplicity = 1

C	-2.25021	0.65575	-1.02591
H	-1.31338	1.24767	-1.04086
H	-3.01142	0.96616	-1.76963
O	1.06184	0.64796	-0.31805
O	-2.41887	-0.27774	-0.27467
C	1.84643	-0.4175	0.20889
H	0.6302	0.3178	-1.12137
H	2.65558	-0.71161	-0.47489
H	2.29648	-0.04969	1.13462
H	1.23948	-1.30432	0.4406

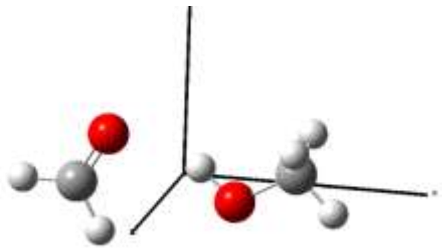

<u>Pathway B2</u>

B2-R-631gd

Charge = 0 Multiplicity = 1

C	-1.87708	-1.85782	-0.00929
H	-1.64911	-1.39512	0.94497
H	-1.60453	-1.28355	-0.88909
O	1.5596	-0.15322	0.04985
O	0.28585	-0.0425	-0.05582
C	-2.87866	-2.91309	-0.09788
H	1.83684	-0.12441	-0.88514
H	-2.21553	-3.83166	-0.12093
H	-3.50995	-3.02566	0.78683
H	-3.45805	-2.9287	-1.02411

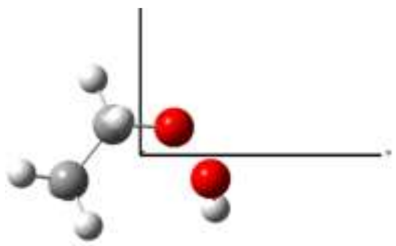

B2-TS-631gd

Charge = 0 Multiplicity = 1

C	-0.77346	0.85278	0.40638
H	-0.92311	0.91345	1.46413
H	-0.73718	1.86434	0.0595
O	1.59635	-1.10522	0.56107
O	0.80598	-0.43523	-0.25675
C	-1.73733	-0.12143	-0.37846
H	2.06074	-1.78709	0.07015
H	-0.40925	-0.93416	-0.75033

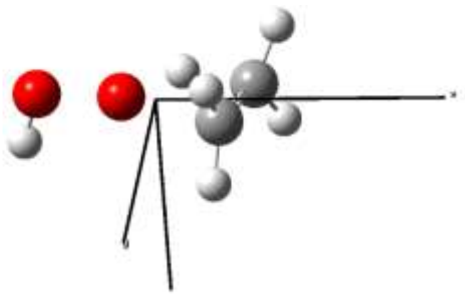

H	-2.46332	-0.64251	0.21001
H	-2.2774	0.30638	-1.19712

B2-P-631gd

Charge = 0 Multiplicity = 1

C	-3.11405	-0.72003	1.26219
H	-3.86211	-0.15039	1.77289
H	-3.03306	-1.77298	1.43438
O	2.87127	-0.2736	-0.51767
O	1.12325	0.00924	-0.10347
C	-2.26918	-0.10791	0.39729
H	2.46997	0.58444	-0.36173
H	1.15144	-0.63055	0.61171
H	-2.35017	0.94504	0.2251
H	-1.52112	-0.67755	-0.11341

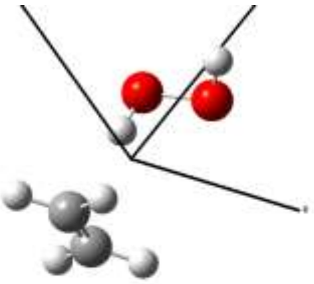

<u>Pathway C1</u>

C1-R-631gd

Charge = 0 Multiplicity = 1

C	-0.79885	0.61025	0.30949
H	-0.50991	0.67159	1.36782
H	-1.34091	1.54429	0.0641
O	1.61914	-0.70989	0.20024
O	0.35251	0.62471	-0.505
C	-1.62861	-0.61825	-0.01699
H	1.46941	-1.16234	-0.65424
H	-1.03836	-1.51366	0.20078
H	-2.54207	-0.64978	0.58738
H	-1.90821	-0.62602	-1.0753
H	2.0194	1.69875	0.65159
H	1.46242	1.48441	0.21194

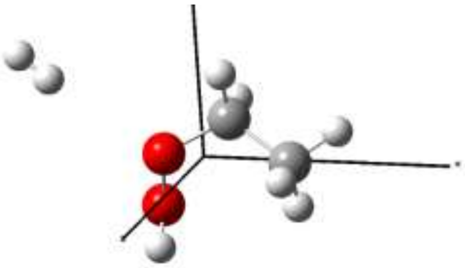

C1-TS-631gd

Charge = 0 Multiplicity = 1

C	0.18165	1.222	0.45806
H	0.60161	1.09649	1.43416
H	0.40707	2.20343	0.09629
O	0.87182	-1.32174	-0.09589
O	0.73749	0.24771	-0.4289
C	-1.34584	1.03936	0.52904
H	0.62595	-2.01458	-0.71324
H	-1.57127	0.05793	0.89081
H	-1.76175	1.76838	1.1927
H	-1.7658	1.16488	-0.44706
H	1.89353	-0.9536	-0.5247
H	1.69702	0.01579	-0.48271

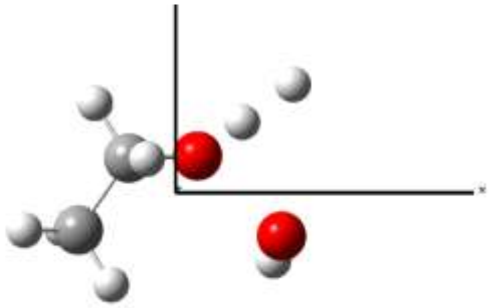

C1-P-631gd

Charge = 0 Multiplicity = 1

C	-0.81578	0.63108	0.31398

H	-0.49261	0.62351	1.36437
H	-1.42618	1.5304	0.14546
O	1.69712	-0.73832	0.24893
O	0.31117	0.68948	-0.54153
C	-1.62227	-0.61784	-0.02009
H	1.39301	-0.97524	-0.64694
H	-1.03419	-1.51459	0.1937
H	-2.5338	-0.64361	0.58686
H	-1.91004	-0.62367	-1.07669
H	2.10959	1.10208	0.64299
H	1.05065	1.11846	-0.06535

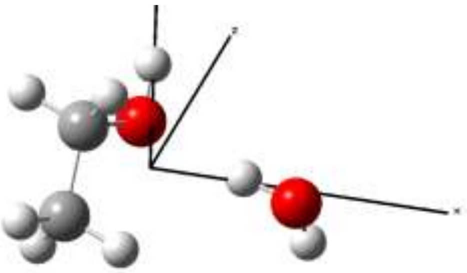

<u>Pathway C2</u>

C2-R-631gd

Charge = 0 Multiplicity = 1

C	-0.48309	0.71585	0.23409
H	-0.29886	0.78171	1.31382
H	-0.52769	1.73462	-0.16995
O	0.97368	-1.08714	0.24667
O	0.67322	0.18067	-0.4103
C	-1.75922	-0.05811	-0.06838
H	0.85079	-1.69603	-0.50337
H	-1.68878	-1.0798	0.31658
H	-2.61929	0.42566	0.40863
H	-1.94261	-0.09908	-1.14778
H	3.57364	1.7981	0.1056
H	2.93151	1.44013	-0.00877

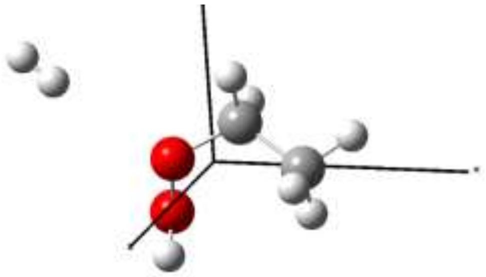

C2-TS-631gd

Charge = 0 Multiplicity = 1

C	-0.82338	-0.35013	-0.1362
H	-0.70055	-0.33735	0.92664
H	-1.10484	0.64366	-0.4156
O	2.2735	-0.73065	-0.12168
O	1.22472	-0.55115	-0.90285
C	-2.29813	-0.77267	-0.00134
H	2.07041	-1.39736	0.53851
H	-2.36659	-1.63086	0.63404
H	-2.86212	0.0314	0.42328
H	-2.69034	-1.01211	-0.96764
H	0.50715	-1.56508	-1.41874
H	-0.29253	-1.5282	-1.15133

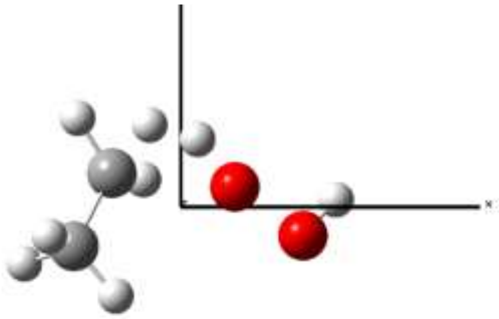

C2-P-631gd

Charge = 0 Multiplicity = 1

C	0.5167	0.79313	0.15331
H	0.61345	0.04729	-0.60779
H	1.4128	0.82524	0.73717
H	0.35295	1.74771	-0.30151
C	-0.67656	0.44647	1.06301
H	-0.5128	-0.50811	1.51784
H	-0.7733	1.1923	1.82412
H	-1.57265	0.41435	0.47916
O	0.25299	-2.17218	-0.87035
O	-0.83087	-2.39044	-0.14925
H	0.75901	-1.45643	-0.47889
H	-1.3369	-3.10619	-0.54071

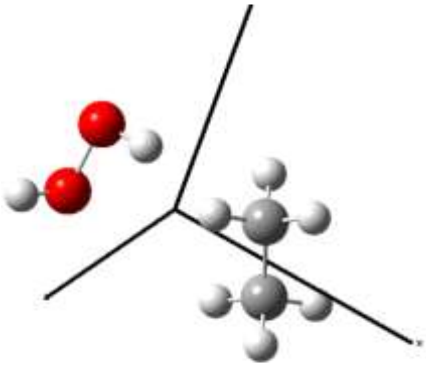

Pathway C3

C3-R-631gd

Charge = 0 Multiplicity = 1

C	1.23029	-0.64794	0.30384
H	0.86671	-0.71095	1.34248
H	1.83724	-1.56563	0.11668
O	-0.90498	1.18818	-0.1136
O	0.22175	-0.69334	-0.61964
C	2.12553	0.56855	0.08489
H	-0.79428	2.17068	-0.14257
H	1.48642	1.44727	0.24337
H	2.9684	0.59148	0.78364
H	2.50108	0.59123	-0.94235
O	-2.15105	-0.53249	0.3771
H	-1.24808	-0.76615	0.04388
H	-2.58504	-0.21036	-0.42871

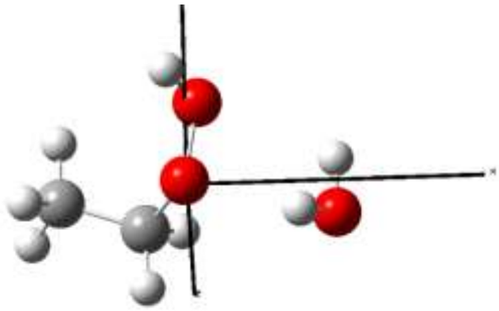

C3-TS-631gd

Charge = 0 Multiplicity = 1

C	0.60422	1.19699	-0.09762
H	0.16101	1.47095	-1.03219
H	0.77317	2.07619	0.48835
O	-1.10982	-1.24903	-0.27945
O	-0.27494	0.31628	0.60688
C	1.94575	0.48574	-0.35462
H	-0.94324	-1.42556	-1.20826
H	1.7768	-0.39346	-0.94059
H	2.60358	1.14474	-0.88176
H	2.38896	0.21178	0.57994
O	-2.72437	-0.22268	0.27253
H	-1.664	0.60929	0.75769
H	-3.36278	-0.60581	0.87853

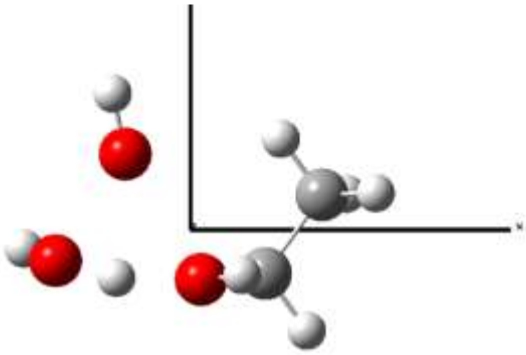

C3-P-631gd

Charge = 0 Multiplicity = 1

C	1.23763	-0.67534	0.31506
H	0.85703	-0.67814	1.34764

H	1.88148	-1.56658	0.20929
O	-1.00511	1.18118	-0.08181
O	0.20662	-0.78453	-0.62036
C	2.1009	0.56482	0.0754
H	-1.41703	2.01418	0.26104
H	1.4863	1.46137	0.2202
H	2.94871	0.5915	0.77085
H	2.48642	0.57541	-0.94969
O	-2.02408	-0.39619	0.33384
H	-0.65929	-0.79969	-0.16884
H	-2.53845	-0.29262	-0.49219

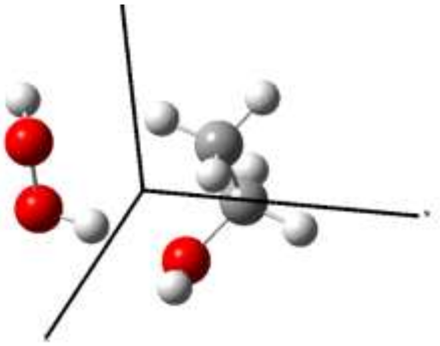

Pathway D1

D1-R-631gd

Charge = 0 Multiplicity = 1

C	1.70353	0.0229	-0.47194
H	0.98668	-0.64074	-0.93869
H	2.3826	0.37119	-1.26187
O	-0.5574	1.14737	-0.11807
O	1.02789	1.20928	-0.01277
C	2.4397	-0.71493	0.6463
H	-0.75189	1.58039	0.73561
H	1.75019	-0.99984	1.44759
H	2.87178	-1.63317	0.2305
H	3.25065	-0.11852	1.07615
C	-1.44718	-0.47294	-0.03397
O	-2.402	-0.20167	0.6123
O	-0.7515	-1.18991	-0.66804

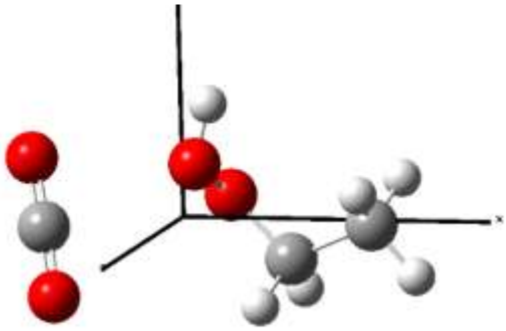

D1-P-6-31gd

Charge = 0 Multiplicity = 1

C	2.52355	-0.21149	0.46021
H	-1.05107	-0.16546	-0.42359
H	2.69225	-0.04322	1.54752
O	-2.29362	1.55117	0.04799
O	1.88016	-1.17495	0.0768
C	3.07047	0.86473	-0.44676
H	-3.03195	2.16804	0.18861
H	3.15804	0.47653	-1.46547
H	2.35276	1.69771	-0.46064
H	4.03632	1.24931	-0.10718
C	-2.85843	0.31961	-0.13921
O	-4.03726	0.09755	-0.04978
O	-1.92739	-0.58406	-0.44364

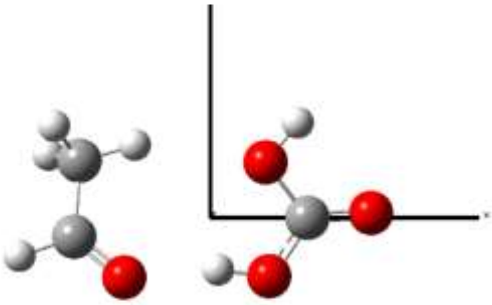

Pathway E1

E1-R-631gd

Charge = 0 Multiplicity = 3

C	-1.85278	0.55759	0.02204

H	-2.32446	1.241	0.73965
H	-2.00829	0.95937	-0.98925
O	0.27513	-0.08253	-0.6426
O	-0.47257	0.65441	0.37034
C	-2.41181	-0.85219	0.15356
H	0.75888	0.65509	-1.05606
H	-2.26995	-1.22732	1.17226
H	-3.48427	-0.85739	-0.07426
H	-1.90823	-1.53083	-0.54042
C	3.83938	-0.20429	0.18654
H	4.07864	0.81423	0.50558
H	2.75585	-0.31832	0.09493
H	4.31693	-0.4051	-0.77697
H	4.21565	-0.9125	0.92985

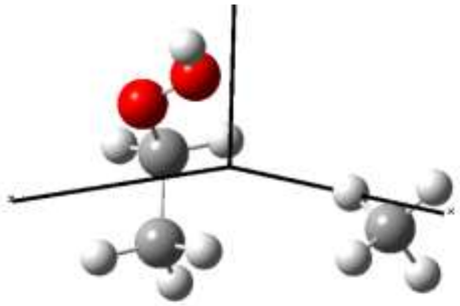

E1-TS-631gd

Charge = 0 Multiplicity = 3

C	-1.82932	-0.00502	-0.13914
H	-2.71984	0.16639	-0.76379
H	-2.0811	-0.62318	0.73357
O	0.84602	-0.53033	-0.91433
O	-0.83134	-0.61321	-0.86978
C	-1.27582	1.35201	0.37182
H	1.13124	-0.94479	-1.75142
H	-0.98161	1.98627	-0.46866
H	-2.06663	1.85404	0.9385
H	-0.41228	1.16944	1.01313
C	3.16581	0.52522	-0.15738
H	2.02893	-0.1198	-0.56769
H	3.76069	-0.07764	0.53428
H	3.77486	0.80355	-1.02223
H	2.82595	1.43261	0.35003

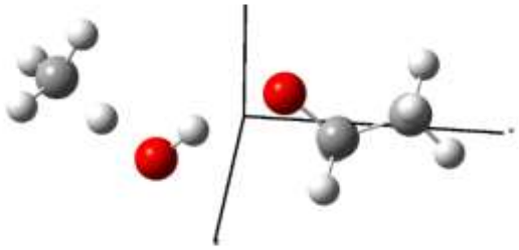

E1-P-631gd

Charge = 0 Multiplicity = 3

C	2.2253	-0.4536	0.29664
H	2.57221	-0.01006	-0.66388
H	3.12596	-0.4539	0.93929
O	-1.62432	1.26138	-2.24114
O	1.30249	0.45493	0.7485
C	1.68488	-1.86355	0.05135
H	-0.95359	1.46911	-1.57033
H	0.80203	-1.81967	-0.59396
H	2.44391	-2.49276	-0.42723
H	1.39964	-2.33412	0.99835
C	-3.30709	0.39013	0.41886
H	-4.14169	1.03392	0.16139
H	-2.36975	0.89041	-1.70822
H	-2.70766	0.65479	1.28341
H	-3.36183	-0.65735	0.1409

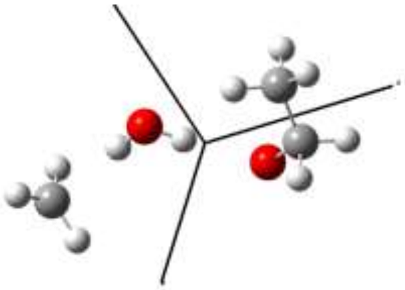

Pathway E2

E2-R-631gd

Charge = -1 Multiplicity = 2

C	2.38152	-0.7889	-0.1249
H	2.31174	-1.71761	0.45509
H	2.84363	-1.01846	-1.09691
O	0.77534	0.80045	-1.1207
O	0.94832	-0.44095	-0.32543
C	3.15159	0.31018	0.60443
H	-0.01236	1.1838	-0.58995
H	2.84317	0.37651	1.65344
H	4.24167	0.17878	0.56308
H	2.88981	1.25405	0.1161
S	-2.60467	-0.19057	0.55263
O	-3.56062	-0.89979	-0.40847
O	-2.47256	1.35243	0.32083

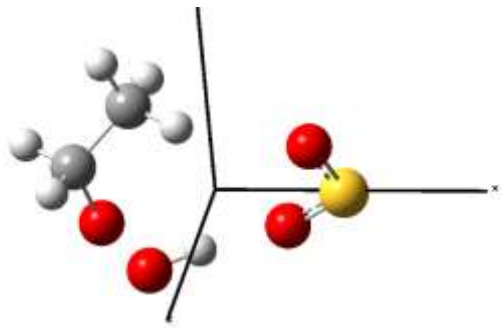

E2-TS-631gd

Charge = -1 Multiplicity = 2

C	-1.42506	0.50365	0.37545
H	-2.27897	0.94457	-0.095
H	-1.63553	-0.51879	0.61042
O	0.68344	-0.62134	-1.2119
O	0.25417	0.60033	-0.95567
C	-1.09874	1.27121	1.67005
H	0.94854	-1.04577	-0.39265
H	-0.88827	2.29365	1.43508
H	-1.9364	1.22298	2.33406
H	-0.24483	0.83029	2.1405
S	2.09233	1.92412	0.1138
O	2.40543	3.09161	-0.72206
O	3.32998	1.05303	0.25135

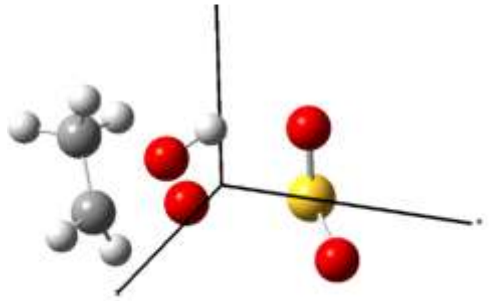

E2-P-631gd

Charge = -1 Multiplicity = 2

C	3.33679	-2.02065	0.28131
H	2.93519	-2.84887	0.85466
H	3.21276	-2.07109	-0.79464
O	0.63794	0.81791	-1.11697
O	0.34579	-0.36168	-0.31106
C	3.63588	-0.7106	0.92919
H	-0.02422	1.43719	-0.69797
H	3.28806	-0.68408	1.96904
H	4.71856	-0.47973	0.94452
H	3.12667	0.0963	0.38723
S	-1.36197	-0.19005	0.4738
O	-2.3588	-0.89469	-0.39225
O	-1.43	1.32277	0.34895

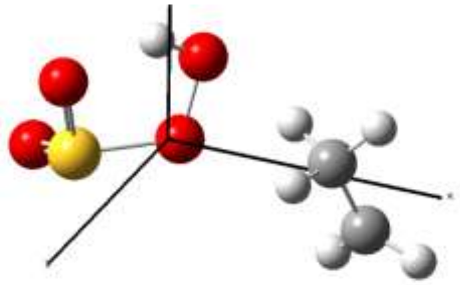

Pathway F1

F1-R-631gd

Charge = 0 Multiplicity = 1

C	1.2495	-0.66331	0.29323
H	0.85297	-0.75523	1.32098
H	1.80288	-1.60605	0.08858
O	-0.88565	1.04133	-0.14811
O	0.25422	-0.60833	-0.659
C	2.20918	0.5166	0.18842
H	-0.68517	2.01631	-0.2275
H	1.63505	1.43188	0.376
H	3.02489	0.45226	0.91812
H	2.63283	0.57658	-0.81927
N	-2.38259	-0.38964	0.32078
H	-1.53233	-0.84293	-0.01215
H	-3.16448	-0.43096	-0.31835
H	-2.62157	-0.55901	1.28843

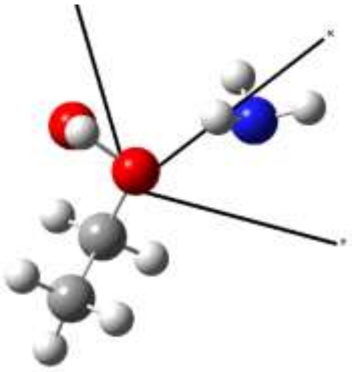

F1-TS-631gd

Charge = 0 Multiplicity = 1

C	-0.72192	-0.42092	0.08073
H	-0.77441	-0.34595	-0.98535
H	-0.88436	-1.43667	0.37528
O	1.35362	1.74006	-0.12806
O	0.56915	0.0037	0.52543
C	-1.80295	0.47583	0.71224
H	1.59329	2.43443	0.49
H	-1.64051	1.49159	0.41769
H	-2.76899	0.15812	0.37949
H	-1.75045	0.40087	1.77832
N	2.72685	0.39	-0.74984
H	1.76512	-0.51288	-0.09732
H	3.64597	0.44977	-0.3604
H	2.86671	0.29001	-1.73495

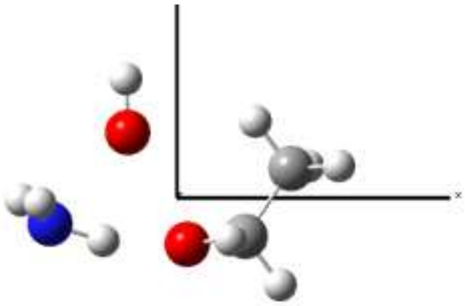

F1-P-631gd

Charge = 0 Multiplicity = 1

C	1.24792	-0.70016	0.29441
H	0.85868	-0.73816	1.33708
H	1.87845	-1.61488	0.19433
O	-1.05105	1.09018	-0.08989
O	0.25747	-0.73688	-0.65766
C	2.1847	0.5125	0.17545
H	-1.42744	1.99338	0.09775
H	1.60549	1.42975	0.34333
H	3.00052	0.46451	0.90921
H	2.6164	0.56445	-0.83025
N	-2.17357	-0.29688	0.24933
H	-1.23444	-0.79414	-0.0941
H	-2.96285	-0.24134	-0.38686
H	-2.41066	-0.30699	1.23635

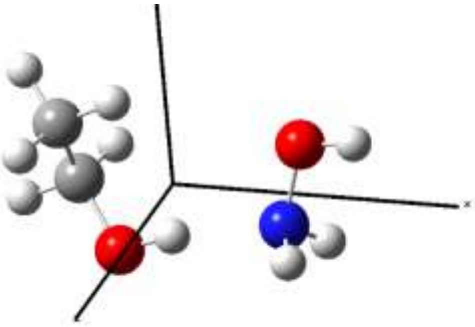

Pathway F2

F2-TS-631gd

Charge = 0 Multiplicity = 1

C	0.25351	-0.73128	-0.03646
H	-0.3461	-1.12767	0.75616
H	0.20935	-1.38822	-0.87989
O	-0.65626	1.96729	0.96847
O	-0.24117	0.55749	-0.40965
C	1.7128	-0.60318	0.43858
H	-0.20287	2.81286	0.93612
H	1.75697	0.05376	1.28201
H	2.08295	-1.5675	0.71782
H	2.31242	-0.20679	-0.35403
N	-2.20283	0.88014	-0.74624
H	-2.46794	1.25468	-1.63474
H	-1.9651	1.8275	0.35785
H	-2.83475	0.12209	-0.58486

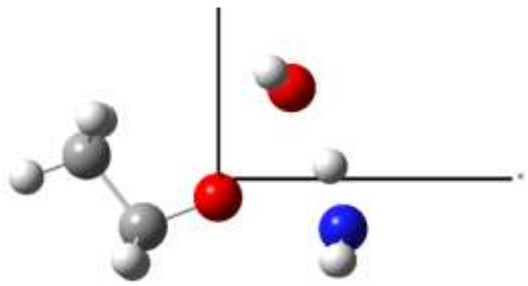

F2-P-631gd

Charge = 0 Multiplicity = 1

C	1.42994	-1.19738	0.67767
H	1.63371	-1.83791	1.55116
H	1.68612	-1.75562	-0.23889
O	-1.06654	2.16651	-1.84684
O	0.13672	-0.65708	0.67326
C	2.37172	0.05482	0.77379
H	-0.64021	2.214	-2.71356
H	2.14644	0.63082	1.67374
H	3.40739	-0.30419	0.81321
H	2.24924	0.70008	-0.09986
N	-1.70173	-1.11297	0.20334
H	-1.65645	-1.75842	-0.59122
H	-1.54902	1.31537	-1.88864
H	-1.62288	-1.68925	1.04711

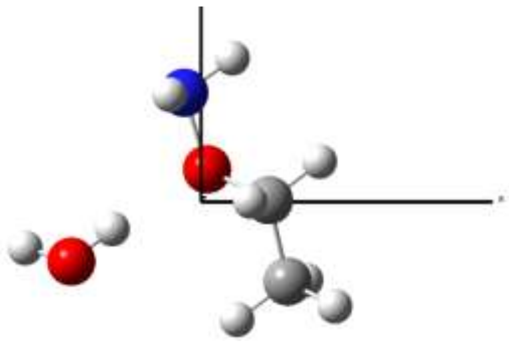

Pathway G1

G1-R-631gd

Charge = 0 Multiplicity = 1

C	2.98292	0.00596	0.01936
H	3.28461	-0.37014	1.00524
H	3.81851	-0.13545	-0.67733

O	0.95515	-0.95063	0.50652
O	1.97763	-0.84937	-0.5201
C	2.56336	1.46891	0.07843
H	0.91647	-1.91917	0.60405
H	1.73296	1.61249	0.77638
H	3.40335	2.08664	0.4171
H	2.25436	1.81867	-0.91202
C	-1.3649	-0.15566	-0.00218
H	-0.77148	0.75437	-0.03229
H	-1.1442	-0.79126	-0.85613
H	-1.22046	-0.67941	0.93674
Cl	-3.1301	0.30213	-0.06297

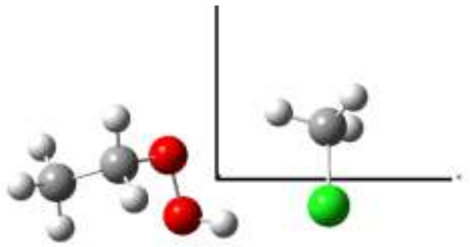

G1-TS-631gd

Charge = 0 Multiplicity = 1

C	-1.58193	-1.3574	1.30133
H	-1.7826	-1.03331	2.30113
H	-1.06296	-2.29276	1.32698
O	-3.77555	0.2151	0.54754
O	-2.81519	-1.51576	0.595
C	-0.71194	-0.30709	0.5861
H	-3.25805	0.88087	1.00637
H	-1.23092	0.62828	0.56045
H	0.21084	-0.18859	1.11461
H	-0.51128	-0.63117	-0.4137
C	-5.68092	0.00221	1.50945
H	-5.71633	-1.025	1.212
H	-5.23871	0.27323	2.44535
H	-6.08771	0.7584	0.87102
Cl	-8.00822	-0.25777	2.68438

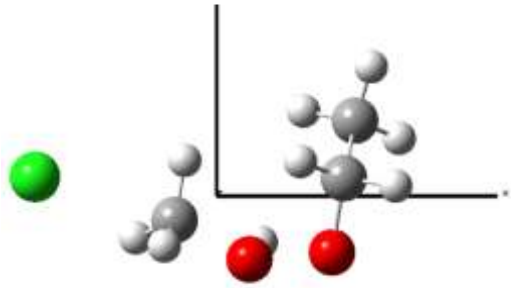

G1-P-631gd

Charge = 0 Multiplicity = 1

C	3.03886	-0.03536	0.07383
H	3.3733	-0.36057	1.07502
H	3.92588	-0.16384	-0.58734
O	0.45065	-0.75025	0.51068
O	2.12832	-0.90618	-0.48184
C	2.62903	1.43871	0.07687
H	0.45548	-1.72369	0.46251
H	1.80717	1.60279	0.78205
H	3.46922	2.06877	0.38857
H	2.30594	1.7543	-0.92071
C	-0.51451	-0.2215	-0.32307
H	-0.30496	0.83581	-0.46809
H	-0.62265	-0.76618	-1.26322
H	-1.60627	-0.22869	0.17358
Cl	-3.31707	0.26509	0.05626

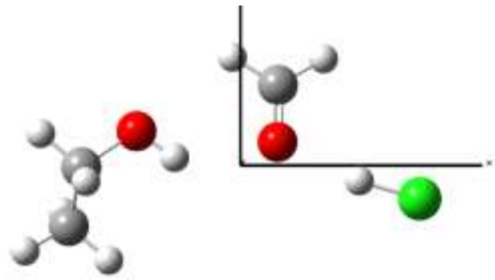

Pathway G2

G2-R-631gd

Charge = 0 Multiplicity = 1

C	2.4266	-0.7026	-0.14113
H	2.29686	-1.50521	0.59444
H	3.02394	-1.08617	-0.97675
O	0.27156	-0.14666	0.34568
O	1.16327	-0.42811	-0.75161
C	3.06861	0.53056	0.47901
H	-0.49226	-0.73966	0.15752
H	2.45244	0.92123	1.29381
H	4.05361	0.27651	0.88716
H	3.19996	1.31617	-0.27297
C	-1.07851	1.60935	-0.31233
H	-1.0361	1.96877	0.70757
H	-0.14493	1.50709	-0.84796
H	-1.94035	1.89721	-0.89778
Cl	-2.50088	-0.36164	0.15832

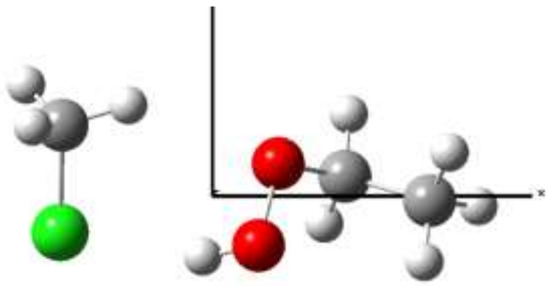

G2-TS-631gd

Charge = 0 Multiplicity = 1

C	0.79505	-0.66721	-0.54823
H	0.33573	-1.63161	-0.48615
H	1.81699	-0.77735	-0.84557
O	-1.85449	0.28469	-1.22742
O	0.10152	0.12993	-1.51181
C	0.73217	0.02088	0.82807
H	-2.14163	-0.20736	-0.45474
H	-0.28977	0.13102	1.1254
H	1.25111	-0.57559	1.54907
H	1.19149	0.98528	0.76599
C	-2.67468	-0.55675	-3.07693
H	-3.70543	-0.42757	-2.82048
H	-2.84251	0.07745	-3.92223
H	-2.86112	-1.608	-3.14765
Cl	-0.12918	-0.87599	-3.71013

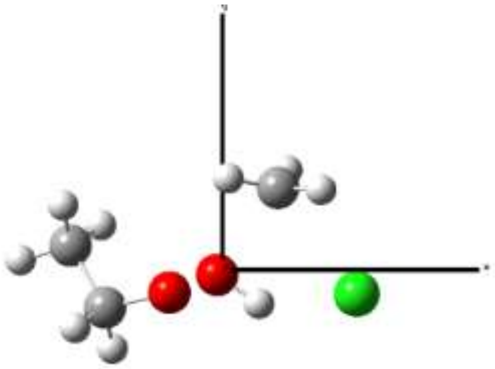

G2-P-631gd

Charge = 0 Multiplicity = 1

C	2.39166	-0.69367	-0.11597
H	2.258	-1.49131	0.62354
H	2.96249	-1.09737	-0.96122
O	0.15836	0.00503	0.24978
O	1.12916	-0.42044	-0.75928
C	3.06138	0.53377	0.47892
H	-0.76683	-0.56727	0.14228
H	2.46436	0.94151	1.29999
H	4.04495	0.26299	0.87866
H	3.19999	1.31079	-0.28001
C	-0.76563	1.58822	-0.276
H	-1.05986	1.95225	0.70132
H	0.14588	1.99206	-0.70286
H	-1.57662	1.35522	-0.95261

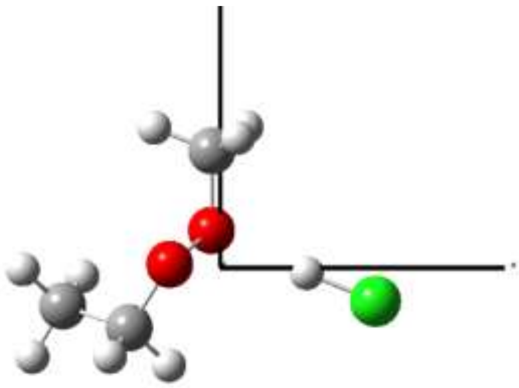

Cl	-2.53387	-0.43441	0.18162

Pathway H1

H1-R-631gd

Charge = 0 Multiplicity = 3

C	-1.62758	-0.49097	0.51298
H	-1.85778	-1.53046	0.79166
H	-1.07557	-0.01208	1.33169
O	-0.06492	1.50741	-0.69922
O	-0.8831	-0.45752	-0.65919
C	-2.9157	0.30658	0.24194
H	0.26744	1.15074	-1.54409
H	-3.46896	-0.12891	-0.59541
H	-3.55175	0.27932	1.13355
H	-2.65695	1.33934	-0.00033
C	1.928	0.96917	0.6889
H	1.96819	1.62683	-0.16715
H	2.88797	0.81942	1.18115
H	1.1206	1.22054	1.36728
Cl	1.51459	-0.88686	0.02505

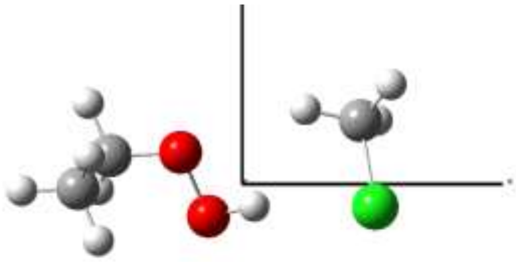

H1-TS-631gd

Charge = 0 Multiplicity = 3

C	-1.55027	-0.49127	1.15858
H	-1.77415	-1.29483	1.8287
H	-1.21318	0.35626	1.71801
O	0.26424	0.58693	-0.98374
O	-0.52532	-0.90048	0.24923
C	-2.81698	-0.11388	0.36826
H	0.12813	0.36422	-1.90758
H	-3.15407	-0.96141	-0.19116
H	-3.5839	0.19231	1.04869
H	-2.59309	0.68968	-0.30186
C	1.65516	0.6437	-0.6566
H	1.97081	0.40443	-1.65059
H	2.66601	0.65115	-0.30585
H	1.59066	1.68676	-0.42691
Cl	1.10262	-1.21259	1.00077

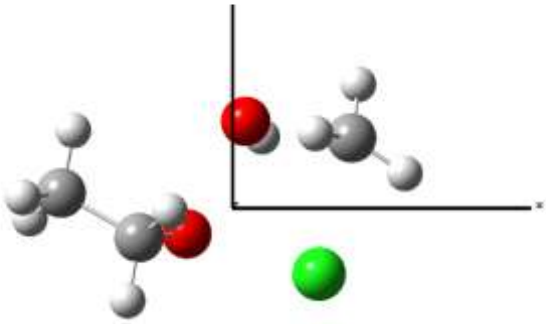

H1-P-631gd

Charge = 0 Multiplicity = 3

C	-1.62173	-0.47801	0.50395

H	-1.82497	-1.5407	0.72201
H	-1.06074	-0.08589	1.36105
O	0.2934	1.45657	-0.46786
O	-0.80331	-0.47129	-0.63031
C	-2.90752	0.30486	0.2461
H	0.61966	1.06759	-1.29885
H	-3.45759	-0.12213	-0.59813
H	-3.5523	0.27626	1.13198
H	-2.66792	1.34724	0.01737
C	1.56479	1.53759	0.51428
H	1.86361	2.58289	0.40642
H	2.38573	0.87469	0.28321
H	1.16017	1.34229	1.50592
Cl	1.43885	-1.08439	-0.03333

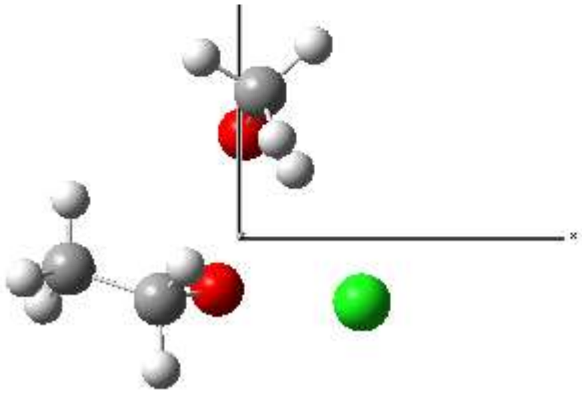

Supplement: Supplementary file 1 — Supplementary Information [file 41598_2020_71881_MOESM1_ESM.pdf]
